# Advanced Pt/Ti_(1−x)_Sn_x_O_2_–C Composite Supported Electrocatalyst with Functionalized Carbon for Sustainable Energy Conversion Technologies

**DOI:** 10.3390/nano15050342

**Published:** 2025-02-22

**Authors:** Cristina Silva, Zoltán Pászti, Khirdakhanim Salmanzade, Dániel Olasz, Erzsébet Dodony, György Sáfrán, Ágnes Szegedi, Zoltán Sebestyén, András Tompos, Irina Borbáth

**Affiliations:** 1 Institute of Materials and Environmental Chemistry, HUN-REN Research Centre for Natural Sciences, Magyar Tudósok Körútja 2, H-1117 Budapest, Hungary; silva.cristina@ttk.hu (C.S.); ksalmanzade5@gmail.com (K.S.); szegedi.agnes@ttk.hu (Á.S.); sebestyen.zoltan@ttk.hu (Z.S.); borbath.irina@ttk.hu (I.B.); 2 Department of Physical Chemistry and Materials Science, Faculty of Chemical Technology and Biotechnology, Budapest University of Technology and Economics, Műegyetem rkp. 3, H-1111 Budapest, Hungary; 3 Institute for Technical Physics and Materials Science, Centre for Energy Research, Konkoly-Thege Miklós út 29-33, H-1121 Budapest, Hungary; olasz.daniel@ek.hun-ren.hu (D.O.); dodony.erzsebet@ek.hun-ren.hu (E.D.); safran.gyorgy@ekhun-ren.hu (G.S.)

**Keywords:** functionalized carbon, mixed oxide–carbon composite, TiSnO_x_, Pt electrocatalysts, Pt–oxide–C triple junction, platinum–tin interactions, CO-tolerance, long-term stability

## Abstract

Sn-doped TiO_2_–carbon composites were identified as promising multifunctional supports for Pt electrocatalysts, in which the oxide component enhances resistance against corrosion and strong metal–support interactions at the Pt-oxide boundary ensure high stability for the Pt nanoparticles. This work is devoted to the study of the influence of preliminary functionalization of the carbon on the properties of Pt/Ti_0.9_Sn_0.1_O_2_–C catalysts. The structural, compositional and morphological differences between the samples prepared using functionalized or unmodified carbon, as well as the effect of carbon pre-modification on the electrocatalytic behavior of the synthesized Pt catalysts, were investigated using TEM, XRD, XPS, nitrogen adsorption and electrochemical measurements. The presence of oxygen-containing functional groups on carbon treated with HNO_3_ and glucose leads to the formation of a homogeneous coating of the carbon with dispersed crystallites of mixed oxide. Elemental mapping revealed the proximity of Sn species with highly dispersed (2–3 nm) Pt particles. Notably, the electrochemical results indicated enhanced activity in CO electrooxidation for both functionalized and unmodified carbon-containing catalysts. An improvement in the 10,000-cycle long-term stability of the catalyst prepared using functionalized carbon was evident compared to the catalyst with untreated carbon or reference Pt/C.

## 1. Introduction

Polymer electrolyte membrane (PEM) fuel cells are the most advanced scalable fuel cell technology, adaptable to a variety of applications from automotive to small-scale power generation [1]. In spite of significant efforts to find more affordable alternatives, Pt/C is still the industry standard electrocatalyst for manufacturing PEM fuel cells, even if it means that achieving cost- or durability targets is difficult.

Due to its high specific surface area, high electrical conductivity and easily modified structure, carbon is one of the most common support materials used for energy storage, sensors, electrocatalysis, etc. [2,3]. However, it is generally recognized that in order to obtain highly dispersed metal centers, ensuring maximum utilization of the catalyst, the supports—in addition to a significant surface area—must have a high number of anchoring sites. In this respect, strong chemical interaction between the support and metal nanoparticles can help in preventing migration, sintering and agglomeration of the active metal during subsequent heat treatments. The stability of catalytic systems containing platinum supported on carbon can be increased by changing the structure of the carbon and the nature of the metal−support interaction. Therefore, chemical modification of the inert carbon surface is a necessary step leading to an increase in the hydrophilicity of carbon, which in turn will lead to high stability of the metallic phase [4,5,6]. However, the strength of the interaction between the active metal and carbon mainly depends on the type of functional groups that serve as metal nanoparticle anchoring sites [7].

Oxygen-containing functional groups (OFGs) are ideal anchors for carbon structures due to the strong affinity between oxygen and transition metals [8]. Depending on the acid/base properties, the surface OFGs of carbon are divided into *acidic* (e.g., carboxyl), *neutral or weakly acidic* (e.g., phenol hydroxyl), and *basic* (carbonyl, quinone) ones [9,10,11]. Despite the existing disagreements in the literature [12], the most widespread opinion remains that an increase in the number of oxygen surface groups of the support, acting as coordination centers for metal cations, significantly increases the dispersion of active metal nanoparticles [13].

It is certain that OFGs of carbon play an important role in various catalytic applications [14,15,16], but due to the complexity of carbon material structures and the diversity of OFG functions, their catalytic role is still not entirely clear. Typically, these groups can be introduced by liquid-phase oxidation treatments with H_2_O_2_, NaOH, (NH_4_)_2_S_2_O_8_ or acids such as HNO_3_, citric acid, etc., and gas-phase oxidation including O_2_, ozone, carbon dioxide, etc. [17,18,19,20,21,22,23]. Moreover, OFGs on the surface of carbon materials can also be formed by the method of electrochemical oxidation, using various acids, bases and salts as electrolytes [9]. As indicated in refs. [24,25], in the process of electrochemical oxidation, OFGs on the surface of carbon materials are formed by a stepwise mechanism.

It is well-known that the type and the number of OFGs govern not only acidity but can change some of the original bulk properties of the carbon supports, such as their texture, morphology, wettability, adsorption properties, electric conductivity, etc. [26,27,28,29,30]. In this respect, for example, it is necessary to take into account the fact that the presence of a large number of OFGs may cause a decrease in conductivity. In addition, by studying different Pt–carbon systems, Willinger et al. [31] demonstrated that the stability of the graphitic carbon support can be seriously damaged by the presence of a large number of functional groups. Thus, the identification of the properties of the sample corresponding to the optimal chemical and morphological composition of the surface can serve as a guide in choosing the type of modifications to be performed [32]. Despite the large difference in platinum content, the results of fundamental research related to the influence of functionalization treatment of carbon are equally applicable to both catalysis and electrocatalysis [33,34,35].

In spite of the significant amount of knowledge about functionalized carbon as electrocatalyst support, it appears that functionalization alone is not enough to rectify the key problems of the Pt/C fuel cell electrocatalysts, namely, their limited oxygen reduction reaction (ORR) activity and sensitivity to corrosion processes, leading to a short fuel cell lifetime [36]. The widespread use of PEM fuel cells could be facilitated by the development of more durable and affordable catalyst systems that would replace the widely used commercial Pt/C electrocatalysts. In this regard, metal oxides with high corrosion resistance and the ability to act as cocatalysts to enhance the catalytic activity may be promising potential support materials for Pt electrocatalysts [37,38,39,40].

Numerous studies show that TiO_2_ [41,42,43,44,45,46,47] and SnO_2_ [48,49,50,51], taking into account both their chemical inertness and stability even under harsh cathodic conditions [52] and the possibility of synthesizing nanostructured materials with a large surface area [53], are very attractive support materials for fuel cells.

Unfortunately, it should be noted that, as a rule, metal oxides have rather low electrical conductivity, which prevents their wide use as support for electrocatalysts. The most commonly used method for enhancing the conductivity of oxide supports is their combination with carbonaceous materials [54,55,56,57].

In the literature devoted to the preparation of carbon-containing composite materials, much attention is paid to elucidating the role of the previous functionalization of carbon. In the synthesis of composite materials, the functionalization of the carbonaceous supports has a dual effect: it promotes both the strong interaction of the support with the active metal, as well as the formation of a homogeneous metal oxide coating on carbon [58]. Thus, after the functionalization of carbon with HNO_3_, a mixture of HNO_3_–H_2_SO_4_ acids or glucose, the formation of a uniform coating of carbon with small TiO_2_ nanoparticles was demonstrated [59,60]. According to Odetola et al. [61], the use of glucose-adsorbed carbon can be regarded as a reliable way to control the size of the deposited TiO_2_ nanoparticles, resulting in a homogeneous coating and improved conductivity of the TiO_2_–C composite. Poh et al. [62] demonstrated higher methanol oxidation activity associated with the smaller size of Pt nanoparticle obtained on catalyst supported on citric acid-modified carbon compared to HNO_3_–H_2_SO_4_-treated carbon materials. The results obtained in ref. [63] showed high ORR activity and stability during accelerated stress tests on Pt/TiO_2_/C catalysts containing glucose-functionalized carbon.

Our approach is the development of oxophilic metal M (M: W, Mo, Sn)-doped TiO_2_-based Pt/Ti_(1−x)_M_x_O_2_–C electrocatalysts [64,65,66]. In our previous studies, the key to improving catalyst performance was the combination of the stabilizing action of oxides with the electrical conductivity of carbon materials with high specific surface area, accompanied by a simultaneous increase in the amount of Pt–oxide–carbon triple junctions.

The interaction between Pt and the oxophilic dopant M (M: W, Mo, Sn) of TiO_2_ results in an improvement in electrocatalytic performance. In our recent study, the development of Ti_(1−x)_Sn_x_O_2_–C (x = 0.1, 0.2 and 0.3) composite support materials using two sol-gel-based preparation routes was explored and compared [66]. The first method (route *A*) was an adaptation of a previously established technique used for the synthesis of Mo-containing Ti_(1−x)_Mo_x_O_2_–C composites, which involved the stepwise introduction of mixed oxide precursors [67]. In this case, the tin precursor was added after prolonged aging of the solution containing TiO_2_-rutile nuclei and carbon. In contrast, the second method (route *B*) involved the simultaneous introduction of tin and titanium precursors at the beginning of the synthesis, which resulted in good mixing of both Sn- and Ti-sol before the addition of the carbonaceous material. This modification in the synthesis method resulted in a more uniform distribution of the Sn and Ti species, leading to an increase in the catalytic activity and stability of the resulting system [66]. A feature of catalysts supported on these composites was the practical independence of catalytic activity from the Ti/Sn ratio due to the presence of a tin oxide-rich overlayer [68]. However, it should be noted that the best result in long-term stability was obtained on a Pt/Ti_0.9_Sn_0.1_O_2_–C catalyst with the lowest tin content (Ti/Sn = 90/10) synthesized by route *B.* Furthermore, we demonstrated that increasing the carbon content in the composites augmented the catalytic activity and long-term stability, rendering them promising for potential use in PEM fuel cells [66].

Thus, the development of an optimal synthesis route of composite support having a uniform coating of the carbon with dispersed crystallites of mixed oxide and complete incorporation of the dopant M, preventing its dissolution, is one of the most important tasks. In the synthesis of Mo-containing composites, we have previously shown that the presence of functional groups on carbon materials contributes to the process of nucleation and growth of the rutile-TiO_2_ phase, which, in turn, affects the formation of a more homogeneous oxide coating on the functionalized carbon, without the characteristic large, nanorod-like mixed oxide crystallites [69]. This microstructure has the maximum density of Pt–oxide–C triple connections after platinum deposition, ensuring good dispersion and stability of the active metal.

The aim of this work is to explore the effect of carbon functionalization on the microstructure and performance of Pt electrocatalysts deposited onto Ti_(1−x)_Sn_x_O_2_–carbon composite supports prepared by the route *B* mentioned above. Based on the beneficial structural effect of the carbon functionalization on the composites with Mo-containing mixed oxide, it is expected that the ideal uniform oxide coating can be even more closely approximated in the case of finely dispersed tin-doped mixed oxide particles. The microstructure of catalyst samples prepared on supports with 50 wt.% unmodified and functionalized Black Pearls 2000 carbon is compared by means of XRD and TEM/STEM/EDS elemental mapping measurements. Their surface chemical properties are assessed by XPS combined with hydrogen exposure experiments in the electron spectrometer. The electrocatalytic properties are determined by standard electrochemical techniques involving long-term stability tests in a three-electrode cell as well as by rotating disk electrode measurements of the activities in the hydrogen oxidation reaction (HOR) and the ORR. The effect of varying the oxide content of the supports is demonstrated in the example of a catalyst deposited onto a support with 75 wt.% functionalized Black Pearls 2000 carbon. In order to identify the effects of the Pt–support interactions on the catalytic functions, correlations are sought between the structural and electrochemical properties of the materials.

## 2. Materials and Methods

### 2.1. Materials

Commercial carbon (Black Pearls 2000, Cabot, Boston, MA, USA) was functionalized using nitric acid (a.r., Molar Chemicals, Halásztelek, Hungary) and glucose (Reanal, Budapest, Hungary). For composite synthesis, the Ti and Sn precursors were titanium isopropoxide (Sigma-Aldrich, St. Louis, MO, USA) and tin (IV) chloride-5-hydrate (Riedel-de Haen, Seelze, Germany); further chemicals for composite synthesis were nitric acid (see above) and ultrapure water (Millipore, Molsheim, France, 18 MΩ cm). H_2_PtCl_6_ × 6H_2_O (Sigma-Aldrich) was used as a Pt precursor compound; moreover, for Pt loading ethanol, ethylene glycol, HCl and NaBH_4_ (all from Molar Chemicals) were used. A solution of Nafion (DuPont™ Nafion^®^ PFSA Polymer Dispersions DE 520, Wilmington, DE, USA), 2-propanol (*i*-C_3_H_5_OH, Molar Chemicals, 99.9 *v*/*v*%, a.r) and ultrapure water (see above) was used to prepare the catalyst ink. All gases (H_2_, N_2_, Ar) were obtained from Linde Gáz Magyarország Zrt. (Budapest, Hungary) with 5.0 purity.

### 2.2. Functionalization of Commercial Carbon

The functionalization of commercial Black Pearls 2000 carbon was performed according to the procedure described in our recent study [69]. In this work, commercial carbon pre-treated in nitrogen at 1000 °C was functionalized by a two-step treatment with HNO_3_ and glucose (details of such treatments are schematically shown in Figure S1 in the Supplementary Materials). This functionalized carbonaceous material will be denoted as FC throughout the paper.

### 2.3. Preparation of Ti_0.9_Sn_0.1_O_2_–C Composite Materials and Pt Electrocatalysts

The synthesis method of Ti_0.9_Sn_0.1_O_2_–C composite support materials with a Ti_0.9_Sn_0.1_O_2_/C ratio of 75/25 based on commercial carbon Black Pearls 2000 was optimized in our previous study [66].

Our work on Mo-containing composites revealed that both functionalization of the carbon component and increasing its amount in the composite improved the homogeneity of the support, resulting in more stable catalysts [67]. Using this knowledge, in the present work, a catalyst with an oxide/carbon = 50/50 mass ratio was prepared using unmodified and FC carbon. Moreover, an FC-containing composite with an oxide/carbon = 25/75 mass ratio was also synthesized.

**Figure 1:**
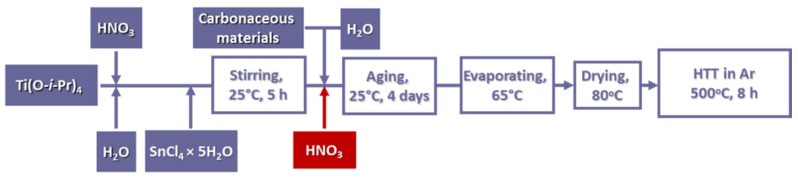
Preparation steps of Ti_0.9_Sn_0.1_O_2_–C composites with Ti_0.9_Sn_0.1_O_2_/C = 50/50 and 25/75 mass ratio; HTT: high-temperature treatment.

As shown in Figure 1, in this synthesis route, a transparent Ti-sol was mixed with the precursor of Sn before the addition of the carbon and the aging step (for more details, see refs. [66,68]). Table 1 summarizes the quantities of the titanium and tin precursors as well as the amount of the carbon materials used in the synthesis of composite materials. The composite with 50 wt.% nominal carbon content on unmodified Black Pearls 2000 is denoted as 50C. 50FC and 75FC indicate composites with 50 and 75 wt.% carbon content on HNO_3_–glucose-functionalized Black Pearls 2000.

Composite-supported electrocatalysts loaded with 20 wt.% Pt were prepared using a modified NaBH_4_-assisted ethylene-glycol reduction-precipitation method (for details, see ref. [68]). The resulting catalysts are denoted as Pt/50C, Pt/50FC and Pt/75FC, following the nomenclature of the supports.

### 2.4. Physicochemical Characterization

The functionalization of the Black Pearls 2000 carbon was investigated by thermogravimetric (TG) and XPS measurements as described in ref. [69]. The characterization of the composite materials and the Pt catalysts deposited onto them was performed by XRD, TEM, XPS and nitrogen adsorption measurements using the same equipment and procedures described in our previous studies [67,68] (see Supplementary Materials for details).

It should be noted that in this study, the high-resolution micrographs were processed by using the ImageJ software [70] (v. 1.54f) and XPS spectra were evaluated with the CasaXPS package [71] (v. 2.3.12, see details in the Supplementary Materials). A quantitative analysis of the XPS data was performed with the XPSMultiQuant package [72,73] (v. 7.83), as described in our previous studies [66]. For identification of the chemical states, the NIST database [74] and ref. [75] were used.

### 2.5. Electrochemical Characterization

The electrochemical characteristics of the Pt catalysts were studied by standard methods involving cyclic voltammetry (CV) and CO_ads_-stripping voltammetry measurements carried out before and after 500-cycle stability test (for more details see refs. [66,67]). In both the short- (500-cycle) and long-term stability test (10,000-cycle), the samples were polarized in cycles between 50 and 1000 mV potential limits at a 100 mV s^−1^ scan rate.

The measurements in 0.5 M H_2_SO_4_ electrolyte were performed in a conventional three-electrode electrochemical glass cell using the same equipment, methods and the composition of the catalyst ink as described in our previous work [67] and in the Supplementary Materials. The working electrode with 10 µg cm^−2^ Pt loading was prepared by depositing the electrocatalysts onto glassy carbon. The cell was equipped with a Pt counter electrode and a reversible hydrogen reference electrode (RHE). All potentials are given on the RHE scale.

The details on the calculation of the electrochemically active Pt surface area (ECSA) from the charge required to oxidize a hydrogen monolayer [76] using conventional baseline correction can be found in our previous studies [64,67] and in the Supplementary Materials. The ECSA loss during the N-cycle stability test (ΔECSA_N_) was calculated from ECSA values in the 1st and Nth cycles (Equation (1)) [67]:

ΔECSA_N_ = {1-(ECSA_N_/ECSA_1_)} × 100% (1)

The catalytic activity of the catalysts was also tested by the rotating disc electrode (RDE) technique in the oxygen reduction reaction and in the hydrogen oxidation reaction as described in our previous study [77] (see Supplementary Materials for details).

The electrochemical performance of the three Pt/Ti_0.9_Sn_0.1_O_2_–C electrocatalysts was compared with commercial 20 wt.% Pt/C (C: Vulcan XC-72, Quintech, Pittsburgh, PA, USA).

## 3. Results and Discussion

### 3.1. Structural and Surface Chemical Characteristics

According to the literature [4], the inert surface of carbon requires suitable chemical modification by which the interaction between the support surface and the metal oxide can be improved. The surface functionalization of carbon was followed by TG and XPS measurements. Thermal analysis provides valuable information about the nature of the introduced functional groups. As shown in Figure S2 in the Supplementary Materials, the functional groups are eliminated in two steps during the thermal decomposition of FC (for more details, see ref. [69] and Supplementary Materials). According to the TG results, the content of oxygen-containing surface functional groups of FC was 25.3 wt.%. XPS confirmed the presence of a high amount of –OH groups due to the glucose functionalization, resulting in surface composition of C = 86.6 and O = 13.4 at.%.

The key assumption of this work was that functional groups on the carbonaceous component of the composite have a discernible structure-directing effect on the formation and growth of the mixed oxide component, which may beneficially influence the functional behavior of the resulting electrocatalysts. Therefore, first, the structure of the catalysts is explored by comparison of XRD, TEM and N_2_ physisorption results for Pt/50C, Pt/50FC and Pt/75FC.

**Figure 2:**
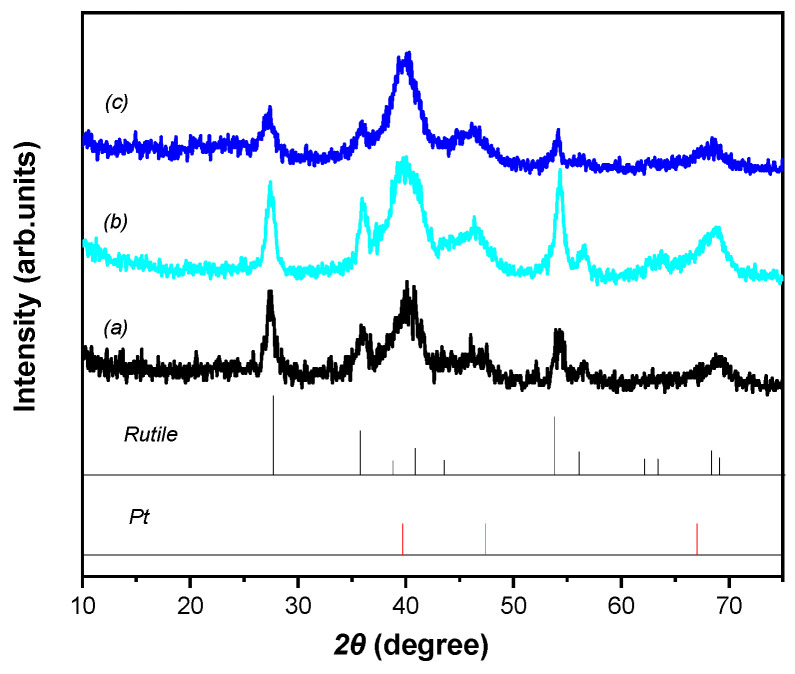
X-ray diffraction patterns of (**a**) the Pt/50C, (**b**) Pt/50FC, (**c**) Pt/75FC electrocatalysts. The expected reflection positions of rutile-TiO_2_ (JCPDS 21-1276) and platinum (JCPDS 04-0802) are shown as bar graphs.

In Figure 2 XRD patterns of the investigated electrocatalysts are shown. The patterns were similar for all samples: only reflections arising from rutile TiO_2_ (JCPDS 21-1276) and face-centered cubic Pt (JCPDS 04-0802) were observed; neither peaks characteristic for other TiO_2_ polymorphs nor features suggesting the presence of SnO_2_ or metallic Sn were seen. The Pt peaks at 2θ values of 39.6°, 47.4°, and 67.1°, arising from the (111), (200) and (220) planes were quite broad, evidencing the formation of rather small particles in all three samples, irrespectively to the support.

**Figure 3:**
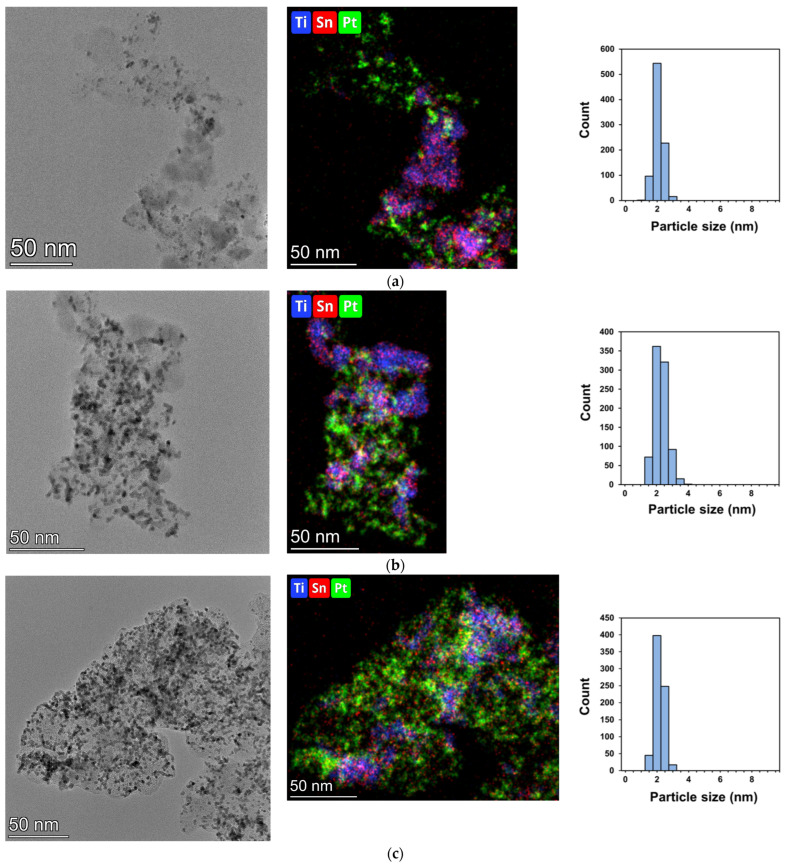
TEM images of the Pt/Ti_0.9_Sn_0.1_O_2_–C electrocatalysts, Ti, Sn and Pt elemental maps and Pt particle size distributions for: (**a**) Pt/50C, (**b**) Pt/50FC and (**c**) Pt/75FC.

**Figure 4:**
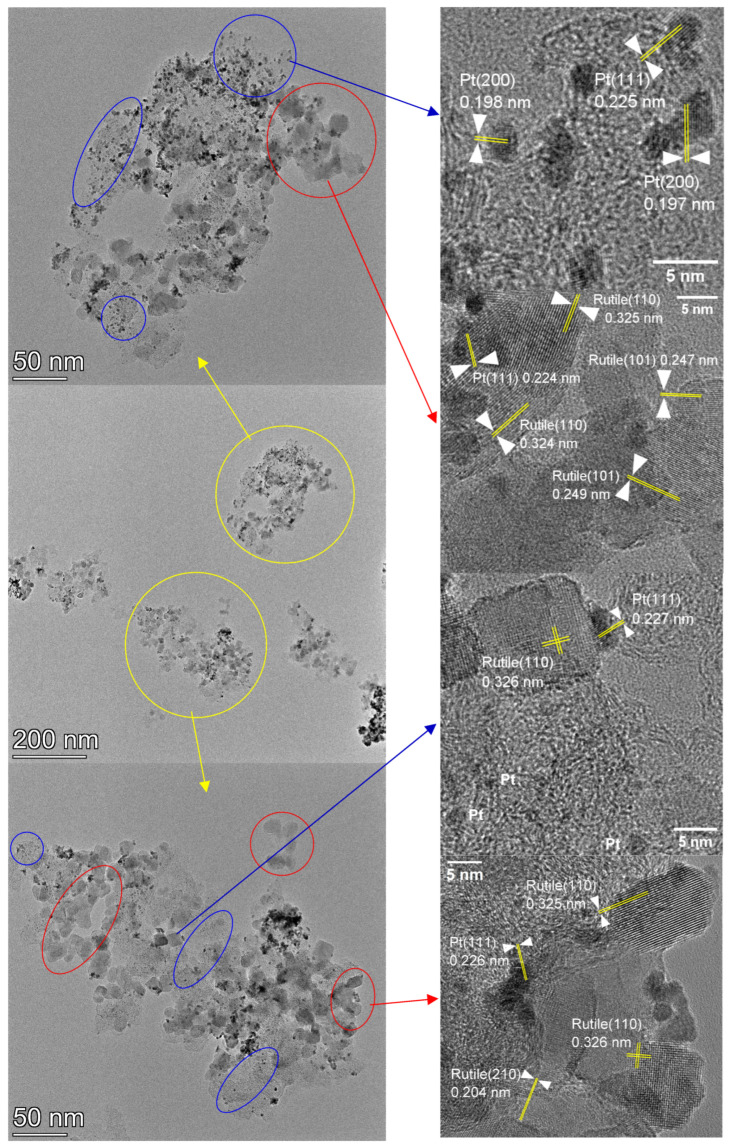
TEM and high-resolution TEM images of the Pt/50C electrocatalyst. Agglomerations of oxide crystals are encircled in red, while Pt/C-like, oxide-lean regions are indicated by blue.

**Figure 5:**
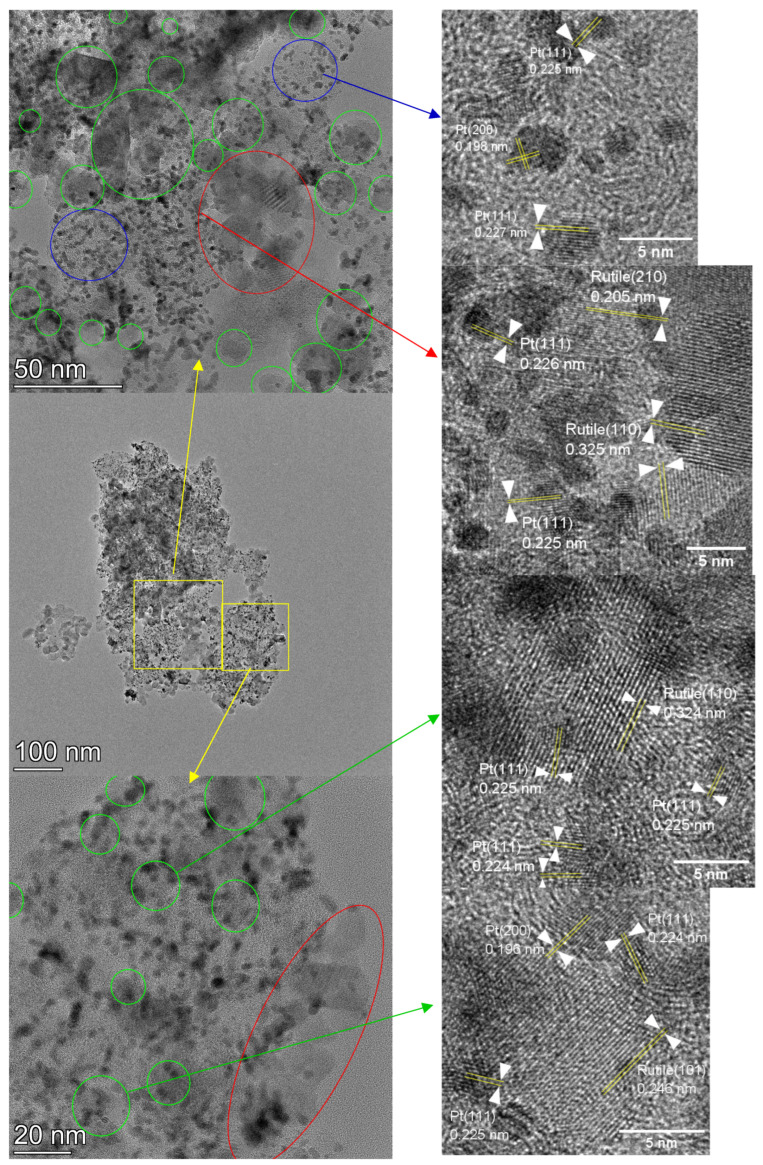
TEM and high-resolution TEM images of the Pt/50FC electrocatalyst. Agglomerations of oxide crystals are encircled in red, Pt/C-like, oxide-lean regions are indicated by blue and individual oxide crystals are highlighted in green.

The microstructure of the electrocatalysts was explored by transmission electron microscopy. Micrographs recorded in bright field mode were completed with scanning TEM images using HAADF imaging and EDS elemental mapping. In Figure 3, characteristic micrographs, elemental maps and Pt particle size distributions for the Pt/50C, Pt/50FC and Pt/75FC electrocatalysts are compared. Pair-wise Ti-Sn and Sn-Pt distributions, along with correlation between the Sn distribution and the HAADF micrographs, are provided in the Supplementary Materials (Figure S3). Details of the microstructure of Pt/50C and Pt/50FC are shown in Figure 4 and Figure 5, respectively. Further bright field micrographs of Pt/75FC, completed by high-resolution images, are given in Figure S4 in the Supplementary Materials.

The morphological elements found in the micrographs of the electrocatalysts prepared on the supports with higher oxide contents were identical to those described in our previous works [66,68]. Namely, relatively large (10–20 nm), usually faceted or somewhat rounded particles giving middle gray contrast surrounded or decorated the carbonaceous components, while small roundish particles with strong dark contrast covered homogeneously the catalyst grains (Figure 3, Figure 4, Figure 5 and Figure S4). Elemental maps in Figure 3 or d-spacing measurements on high-resolution micrographs (Figure 4, Figure 5 and Figure S4) consistently revealed that the 10–20 nm sized faceted, rectangle-shaped particles were oxide crystallites. D-spacing values measured on the high-resolution micrographs of these catalysts indicated that the oxide particles were exclusive of the rutile TiO_2_ polymorph, as evidenced by the prevalence of the rutile (110) (d-spacing around 0.325 nm) or (101) (d-spacing at 0.248 nm) planes. It has to be noted that d-spacing values from rutile SnO_2_ or from anatase or brookite TiO_2_ polymorphs were never found in the micrographs of these samples. The small dark particles were Pt nanoparticles, frequently exhibiting the (111) (d-spacing around 0.226 nm) or the (200) (d-spacing around 0.198 nm) lattice planes. According to their size distribution (Figure 3), essentially identical Pt particles with sizes in the 2–3 nm range were deposited onto all the supports.

However, a clear difference was observed between the distribution of the oxide particles in Pt/50C and Pt/50FC. To emphasize this, in Figure 4 and Figure 5 micrographs taken at increasing magnification from the same region are presented for both samples; the depicted areas were distinct from those shown in Figure 3. According to Figure 4 and Figure 5, low-magnification images demonstrated the large-scale homogeneity of the catalyst grains of both samples; in these micrographs the oxide particles can hardly be noticed. However, at higher magnifications the oxide crystals can be distinguished, resulting in a more structured appearance. At these magnifications, the Pt/50C electrocatalyst (Figure 4) was somewhat inhomogeneous with oxide-rich regions, frequently containing chains or loose aggregates of the oxide (encircled by red) and oxide-lean regions, consisting mainly of Pt and C (encircled by blue). The oxide chains or aggregates were often found around the edge of the carbonaceous component. On the other hand, while the size and shape of the oxide particles in Pt/50FC (Figure 5) were rather similar to those found in the Pt/50C catalyst, they were more homogeneously dispersed over the carbonaceous backbone. In particular, the long oxide chains or the large agglomerations of the oxide particles at the edge of the carbon formations were missing, although smaller aggregations were sometimes detected (encircled by red) and the oxide particles were still frequently seen along the perimeter of the carbon structures. Areas without oxide particles (encircled by blue) were also smaller and less frequent. At the same time, in apparently homogeneous-looking regions, numerous individual oxide crystals were identified. They were less visible than in the case of the sample on unmodified carbon, so they were highlighted by green encircling.

Elemental maps of Pt/50C (Figure 3a and Figure S3a) demonstrated that the distribution of Sn and Ti was highly correlated, although Sn was found in small quantities in the oxide-lean regions. Pt was more or less evenly distributed over the surface of the support, so both Pt–carbon and Pt–oxide–carbon connections were numerous in this catalyst. High-resolution micrographs (Figure 4) confirmed the presence of the Pt particles in the immediate vicinity of the oxide crystallites.

In the case of Pt/50FC, Ti was still concentrated in the oxide particles (Figure 3b and Figure S3b). While the oxide particles provided also intense Sn signals, the distribution of tin was not strictly correlated with that of Ti, instead, it more or less homogeneously covered the support (Figure S3b). According to the high-resolution micrographs, no sign of crystalline Sn-containing oxide particles was seen in the regions between the Ti–Sn mixed oxide particles, so Sn indicated by elemental mapping in these areas must have formed very thin and/or small disordered structures. At the same time, Pt particles were found on both the oxide crystallites or around them (Figure 5), providing a high density of Pt–oxide–C triple junctions. The homogeneous presence of Sn on the support ensured that the surface chemical and electrocatalytic properties of the system were governed by the Sn–Pt coupling.

In the case of the Pt/75FC catalyst (Figure S4), lower magnification TEM micrographs were dominated by the Pt nanoparticles, while the oxide particles were less evident. Nevertheless, elemental maps (Figure 3c and Figure S3c), as well as high-resolution micrographs (Figure S4), revealed the quite homogeneous presence of smaller, 5–10 nm oxide particles, often with a roundish or elongated shape (emphasized by the green encircling). According to d-spacing data, the oxide particles were still exclusively of the rutile polymorph. They were frequently closely associated with Pt nanoparticles, which were in the usual 2–3 nm size range. Small agglomerations of a few oxide crystallites were only rarely noticed (red encircling in Figure S4). According to the elemental maps, the distribution of Ti and Sn was uncorrelated: Ti was present only in the oxide particles, while Sn homogeneously covered the entire support. As Pt particles were also homogeneously dispersed, the distribution of Pt and Sn was quite congruent.

In summary, electron microscopy studies indicated a twofold effect of carbon functionalization on the microstructure of the electrocatalysts. Firstly, the association between the oxide particles and the carbonaceous backbone became stronger, resulting in the development of a more homogeneous coverage of the mixed oxide on the carbon, instead of the prevalence of large, loose oxide agglomerations characteristic of the non-functionalized carbon material. Secondly, functionalized carbon interacted with tin more strongly, leading to a quite homogeneous dispersion of tin species over the carbon, while in the case of non-functionalized carbon, it was located almost exclusively in the mixed oxide crystallites. The distribution of Pt was not influenced by the functionalization, which is not surprising as the majority of functional groups were eliminated in the high-temperature treatment process of the composite support preparation. Consequently, the use of functionalized carbon certainly increased the homogeneity of the electrocatalysts and improved the density of the Pt–oxide–carbon triple junctions, along with the extent of the Sn–Pt interactions.

Decreasing the amount of the oxide component of the composite resulted in a significant decrease in the size of the mixed oxide particles, while they were even more homogeneously dispersed on the carbon. At the same time, the homogeneous spreading of tin over the functionalized carbon remained unchanged.

The specific surface area and pore characteristics of the support materials and related Pt electrocatalysts were determined by nitrogen physisorption measurements. The results of the surface area measurements are summarized in Table S1 in the Supplementary Materials. Data in Table S1 indicate that all composites had an appropriate specific surface area (S_BET_) for fuel cell electrocatalyst preparation (>100 m^2^ g^−1^). A comparison of samples with the mixed oxide/carbon ratio of 50/50 presented in Table S1 in the Supplementary Materials showed that the S_BET_, pore diameter and micropore volume were smaller on the 50FC composite in comparison to the 50C ones. Similar results were obtained in the case of the Mo-doped TiO_2_-carbon composite supports synthesized using functionalized and unmodified carbon [67,69]. The reason may be the formation of a more uniform mixed oxide layer on the functionalized carbon compared to the unmodified one. In ref. [78], a significant decrease in the micropore volume of TiO_2_-coated activated carbon compared with an un-coated one was evidenced, which was explained by the filling of pores during the TiO_2_ growth process. The pronounced decrease in the volume of micropores observed on the 50FC composite (see Table S1) can be attributed to the blocking of the micropores of the carbonaceous material by oxide particles. In this regard, the presence of a uniform coating can lead to a significant decrease or even complete loss of surface area inside the pores for nitrogen adsorption. In contrast, if only a few large mixed oxide particles were formed on the external carbon surface, the measured S_BET_ should even increase to some extent, consistent with the additional contribution of the separated species.

At the same time, increasing carbon content resulted in higher S_BET,_ even for the composites prepared with FC. Comparison of the N_2_ adsorption isotherms, indicating structural differences between composites with unmodified and functionalized carbon (see Figure S5 in the Supplementary Materials), was consistent with the results of the TEM investigations.

The surface composition and surface chemical properties of the electrocatalyst samples were determined by XPS. Selected composition data, completed by structural information, are summarized in Table 2.

According to our previous investigations, the relative closeness of the oxide/carbon weight ratio measured by XPS to the nominal value is a characteristic feature of the applied composite preparation method and is related to the more or less homogeneous distribution and comparable dimensions of the oxide and carbonaceous elements of the support [66,68]. Indeed, this quantity is still scattered around the nominal 50:50 value for the systems prepared with 50 wt.% carbon in the composite (Table 2). Nevertheless, the amount of oxide deduced from XPS was consistently higher than the nominal value for both FC-containing supports, which can be explained by the combined effect of the more homogeneously dispersed nature of the oxide crystallites on FC carbon and the spreading of the ionic tin species (see the TEM and elemental mapping results). The high apparent Pt content obtained by XPS can be attributed to the localization of the Pt particles at the outer surface of the support grains.

According to Table 2, the Ti/Sn atomic ratio measured in the catalysts by STEM/EDS was close to the nominal value (around 10:1, while the nominal value is 9:1). At the same time, XPS results indicated that the surface of all catalysts was significantly enriched in tin, resulting in Ti/Sn ratios of 2–3:1. This high surface tin content is a peculiarity of the composite preparation route and is the result of the segregation of a tin oxide overlayer to the surface of the mixed oxide crystallites [66,68], which interrupts the growth of the oxide particles and is responsible for their characteristic faceted appearance [79]. Another contributor to the surface tin excess is the presence of highly dispersed Sn species between the oxide crystallites. Since the Pt nanoparticles were deposited onto this tin-rich overlayer, it is expected that the surface chemistry of the catalysts (and, in turn, their electrocatalytic properties) are dominated by the coupling between Pt and the surface tin species.

In order to obtain an insight into this Sn–Pt interaction, the XPS investigation of the electrocatalysts in their as-*prepared* (air exposed) state was completed by a hydrogen exposure experiment for 1 h at 100 °C in 100 mbar in the high-pressure chamber of the electron spectrometer. As pointed out in our earlier works, during this treatment, Sn in the atomic closeness of Pt is reduced to the metallic state by the spillover of hydrogen activated on the Pt sites; thus, the metallic Sn signal after the hydrogen exposure can serve as a measure of the Sn-Pt coupling [68,80]. In Table 3, chemical state information on Pt, Sn and Ti based on the measured core level binding energies of these components is summarized for the investigated catalysts before and after hydrogen exposure.

According to the data in Table 3, Pt was predominantly metallic even in the as-*prepared* state of the catalysts, as indicated by its 4f_7/2_ binding energy being slightly above 71 eV. The relatively weak ionic contributions in the as-*prepared* state can be attributed to oxidation during storage in air; nevertheless, they completely disappeared after hydrogen exposure. Ti remained completely oxidized during the experiments.

Upon comparison of the Pt 4f and Ti 2p peak positions, an opposite shift (decreasing Pt 4f_7/2_ and increasing Ti 2p_3/2_ binding energies) can be observed after hydrogen exposure for all electrocatalysts. This is regarded as a manifestation of the electronic metal-support interaction, during which electrons are transported from the oxide crystals towards the contacting Pt particles. The electronic structure change of the Pt particles induced by the mentioned charge transfer effect is believed to beneficially modulate their catalytic activity, especially in the ORR [81].

**Figure 6:**
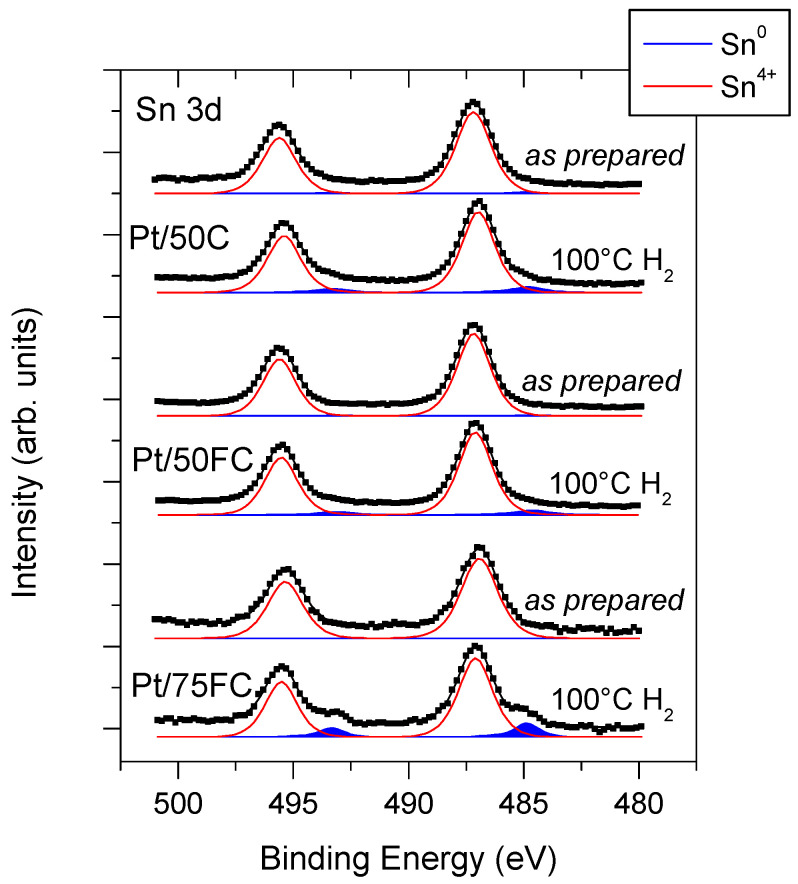
Sn 3d core level spectra of the Pt/50C, Pt/50FC and Pt/75FC electrocatalysts in their as-*prepared* state and after hydrogen exposure.

Sn 3d spectra of the investigated electrocatalysts before and after the hydrogen exposure step are shown in Figure 6. The Sn 3d_5/2_-3d_3/2_ doublet was dominated by a relatively narrow peak pair located around 487.0 and 495.4 eV binding energies, which can be attributed to the Sn^4+^ species [74]. Consistent with this assignment, the Sn M_4_N_45_N_45_ Auger peak was observed around 432.0 eV kinetic energy, resulting in a Sn 3d_5/2_-Sn M_4_N_45_N_45_ Auger parameter value (sum of the Sn 3d_5/2_ binding energy and the Sn M_4_N_45_N_45_ kinetic energy) around 919.0 eV, in agreement with data available for Sn^4+^ [74].

In the as-*prepared* state of the catalysts, only a very weak asymmetry at the low binding energy side of the ionic Sn 3d peaks may point to the presence of more reduced tin species. The very weak doublets needed to model these asymmetries were located around or slightly below 485.0 eV (for the 3d_5/2_ component) and 493.4 eV (3d_3/2_ component), which is characteristic for metallic tin. Importantly, reduced tin components with a 3d_5/2_ binding energy clearly above 485 eV were not observed. As pointed out in [68], the appearance of such “quasimetallic” tin signals in the air exposed catalysts is an indicator of Sn-Pt alloy formation, so significant alloying during Pt loading can be ruled out.

The practically exclusive presence of the Sn^4+^ ions in the as-*prepared* state of the catalysts confirms that the Sn species dispersed on the carbonaceous part of the composite support in the case of the FC carbon component (Figure 3 and Figure S3) are indeed oxidized.

Hydrogen exposure resulted in relatively little but comparable reduction of Sn in the case of the catalysts prepared on supports with 50 wt.% carbon content. The most pronounced reduced tin signals were observed for the Pt/75FC system. The Sn contributions emerging after hydrogen treatment were characterized by a 3d_5/2_ peak slightly below 485.0 eV binding energy, indicating the formation of metallic tin. The development of metallic tin during hydrogen exposure evidences the close coupling between Pt and the surrounding Sn species; the overlapping of the Pt and Sn elemental distributions indeed suggested that such coupling is feasible in all investigated catalysts. According to the present experiment, this coupling is the strongest for the Pt/75FC electrocatalyst; it is expected that its electrochemical properties, especially the CO electrooxidation performance, will reflect this interaction.

### 3.2. Electrochemical Characteristics of the Electrocatalysts

According to the above results, it can be concluded that the functionalization of the carbonaceous part of composite materials leads to a favorable effect on the growth of a more homogeneous mixed oxide coating over the functionalized carbon. Furthermore, the utilization of functionalized carbon can facilitate enhanced interactions between the mixed oxide phase and the carbon backbone, which improves integration within the composite material. This microstructure has an increased density of Pt–oxide–C triple assemblies, which should ensure good stability and activity of the active metal.

**Figure 7:**
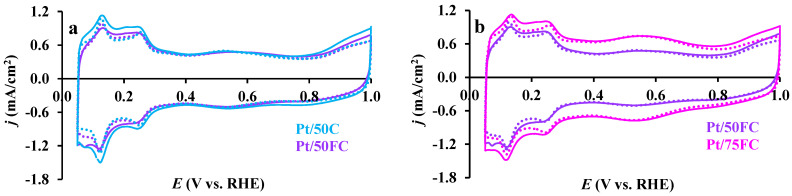
Effect of carbon functionalization (**a**) and increasing of the Ti_0.9_Sn_0.1_O_2_/C ratio in composite support (**b**) on the Pt electrocatalysts performance. Catalysts prepared using unmodified (Pt/50C (■)) and functionalized carbon materials (Pt/50FC (■)) and with 75 wt.% FC content (Pt/75FC (■)). Recorded before (solid curves) and after 500 cycles (dotted curves) of the stability test, sweep rate: 100 mV s^−1^.

The effect of functionalization treatment of carbon (Pt/50C vs. Pt/50FC) and increasing the carbon content in composite from 50 to 75 wt.% (Pt/50FC vs. Pt/75FC) on the electrochemical behavior of the corresponding Pt electrocatalysts is presented in Figure 7. The cyclic voltammograms show the common characteristics of Pt-based electrocatalysts, involving those supported on Ti_(1−x)_Sn_x_O_2_–C composite [66]: (i) adsorption/desorption peaks of underpotentially deposited hydrogen in the range between 50 and 350 mV, and (ii) oxidation/reduction Pt peaks above 800 mV. The difference in the carbon content of composite support materials is mainly visible in the double-layer region: the increase of the carbon content in the composite materials from 50 to 75 wt.% is accompanied by a certain increase in the double-layer capacitance of electrocatalysts, showing wider CV hysteresis (cf. voltammograms presented in Figure 7b for Pt/50FC and Pt/75FC catalysts).

However, as shown in Figure 7a, the functionalization of the carbonaceous part of composite support materials has only a minor effect on the shape of the cyclic voltammograms. This applies primarily to the same size of the double-layer region observed on the Pt/50C and Pt/50FC catalysts (see Figure 7a). According to the literature [82,83], functional groups can significantly enhance the capacitive current in the double-layer region due to their polar nature, promoting a more favorable environment for ion adsorption from the electrolyte, leading to increased charge accumulation in the double-layer. However, it should be noted that in this case, the main goal of carbon functionalization was to create a stable and homogeneous oxide coating over the carbon backbone. Moreover, it is necessary to take into account the fact that high-temperature treatment in Ar at 500 °C was used for the incorporation of tin into the TiO_2_-rutile lattice, which results in partial removal of oxygen-containing functional groups that decompose at a temperature below 500 °C. In addition, in the case of uniform oxide coating over the carbon surface, only a small number of functional groups can come into contact with the electrolyte.

The ECSA values of Pt electrocatalysts calculated from the first cycle (ECSA_1_) are given in Table 4. In addition, the ECSA_1_ values for the reference Pt/C and catalyst with low carbon content in composite with Ti_0.9_Sn_0.1_O_2_/C = 75/25 ratio reported in ref. [66] were also included for comparison. As follows from Table 4, for all Pt/Ti_0.9_Sn_0.1_O_2_–C catalysts, fairly close ECSA_1_ values were obtained around 55.2 ± 3.5 m^2^/g_Pt_, which is at the lower end of the typical range of ECSAs reported for Mo- or Sn-doped mixed oxide–carbon composite supported electrocatalysts in our previous works (60–80 m^2^/g_Pt_ [66,67]).

**Figure 8:**
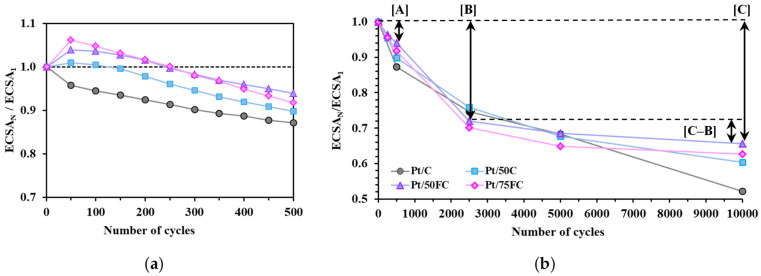
Change of ECSA during 500 (**a**) and 10,000 CV cycles (**b**) for the Pt/50C (■), Pt/50FC (■), Pt/75FC (■) and reference Pt/C (■) electrocatalysts. ECSA_N_/ECSA_1_ values are compared as a function of the cycle number. The ΔECSA_500_, ΔECSA_2500_ and ΔECSA_10,000_ values were labeled as A, B and C, respectively; the change in ΔECSA observed between 2500 and 10,000 cycles was presented as [C−B] (see Table 4 for details).

The changes in the ECSA over 500 and 10,000 CV cycles for the reference Pt/C and three Sn-containing electrocatalysts (Pt/50C, Pt/50FC and Pt/75FC) are shown in Figure 8a and Figure 8b, respectively.

As shown in Figure 8a, the initial small increase in the ECSA during the first 50–150 cycles of polarization is a characteristic feature of the Sn-containing catalysts, which can be interpreted as cleaning of the Pt surface associated with the removal of impurities or oxides that could potentially prevent hydrogen adsorption [68]. Similar behavior was observed on Sn–Pt/C alloy electrocatalysts after contact with air [84]; it was attributed to the blocking of the surface of the metallic particles with a thin SnO_x_ overlayer, resulting in a pronounced decrease in H_2_ adsorption/desorption. However, the low stability of SnO_2_ at high potentials leads to dissolution in acidic conditions [85] and cleaning of the electrode surface from SnO_x_ species [84].

After 150–200 cycles, a slight decrease in ECSA was observed for all catalysts shown in Figure 8a, with the relative ECSA loss increasing in the following order: Pt/50FC ≈ Pt/75FC < Pt/50C < Pt/C, indicating an improvement in the stability of the FC-containing catalysts compared to the unmodified carbon-containing sample (Pt/50C) and the reference Pt/C.

According to the literature [59,61,63] and our previous studies [67,69], one of the most important results of carbon functionalization is the improvement in catalysts’ stability due to the formation of a more uniform oxide coating over the carbon surface. It seems that carbon functionalization modifies the nucleation and growth of the mixed oxide particles; as the carbon component behaves as a solid template, this results in the homogeneous distribution of mixed oxide particles/layers [67,69].

The positive effect of carbon functionalization is confirmed by Figure 8b, and the most stable catalyst in this series of experiments is the Pt/50FC sample with a Ti_0.9_Sn_0.1_O_2_/C mass ratio of 50/50, showing after 10,000 polarization cycles an ECSA loss of ~34% (see Table 4).

**Figure 9:**
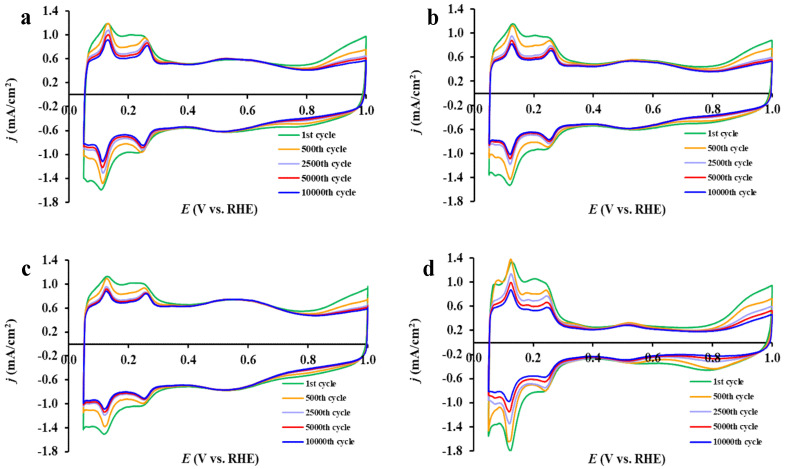
Cyclic voltammograms of the reference Pt/50C (**a**), Pt/50FC (**b**), Pt/75FC (**c**), and Pt/C (**d**) electrocatalysts obtained during 10,000-cycle stability test. Recorded with 100 mV s^−1^ sweep rate, T = 25 °C.

The shape changes of the cyclic voltammograms recorded during the long-term stability test for Sn-containing Pt electrocatalysts are compared with those measured in the reference Pt/C catalyst in Figure 9. As shown in Figure 9, the main changes occurred in the potential range of 0.05–0.35 V (hydrogen adsorption/desorption peaks) and above 0.80 V (Pt oxidation/reduction peaks), demonstrating good stability of the composite supported catalysts in the double-layer potential region (300 < E < 700 mV).

As can be seen from Figure 9, the main difference in the stability of catalysts prepared using FC- and unmodified carbon during 10,000-cycle polarization in the potential range of 0.05–1.00 V appears after 2500 cycles: while FC-containing catalysts showed only a negligible decrease in ECSA after 2500 cycles, the catalysts with unmodified carbon demonstrated more significant changes (cf. cyclic voltammograms in Figure 9b,c with Figure 9a); the most pronounced constant decrease was observed on the reference Pt/C (Figure 9d). The best stability after 2500 cycles was demonstrated by the FC-containing Pt/50FC and Pt/75FC catalysts, which is reflected in lower values of ΔECSA_10,000_ presented in Table 4.

Evidence for these observations is demonstrated in Figure 8b, in which the relative ECSA loss values of the investigated catalysts are shown as a function of the number of stability test cycles. As illustrated in Figure 8b and Table 4, the smallest changes in ΔECSA observed between 2500 and 10,000 cycles ([C−B] values) were obtained on the FC-containing Pt/50FC and Pt/75FC catalysts. According to the result shown in Figure 8b, the highest [C−B] value was obtained on the reference Pt/C catalyst. It should be noted that, as emerges from Table 4, the decrease in carbon content in the composite support materials results in some decrease in the long-term stability, which is reflected in higher [C−B] values (cf. the results obtained on the Pt/25C and Pt/50C samples).

These results are in good agreement with those obtained in our recent study for Mo-containing composite-supported catalysts [67], showing that the homogeneity of the mixed oxide microstructure of catalysts with high functionalized carbon content is the key to obtaining promising catalytic systems. It has been demonstrated [67] that Pt not coupled to mixed oxide is susceptible to severe degradation during potential cycling due to electrocorrosion, which can even lead to the detachment of Pt particles. Thus, the non-uniformity of the oxide structure in the catalysts, where Pt particles can be in contact with areas of carbon not covered with mixed oxide, as well as with regions having stable mixed oxide coatings, can be the reason for lower stability compared to samples with a more homogeneous microstructure.

**Figure 10:**
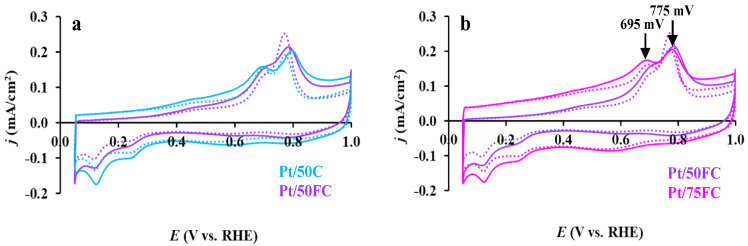
Effect of carbon functionalization (**a**) and increasing of the Ti_0.9_Sn_0.1_O_2_/C ratio in composite support (**b**) on the Pt electrocatalysts performance. Catalysts prepared using unmodified (Pt/50C (■)) and functionalized carbon materials (Pt/50FC (■)) and with 75 wt.% FC content (Pt/75FC (■)). Recorded before (solid curves) and after 500 cycles (dotted curves) of the stability test, sweep rate: 10 mV s^−1^.

The effect of the functionalization treatment of carbon and increase in the Ti_0.9_Sn_0.1_O_2_/C ratio in the composite materials on the CO-electrooxidation activity is demonstrated in Figure 10a and Figure 10b, respectively. CO_ads_ stripping voltammograms recorded before and after 500 cycles of the stability test, presented in Figure 10, exhibit features characteristic of Sn-containing composite supported electrocatalysts [66]. As shown in Figure 10, CO electrooxidation begins at E_CO,onset_ ≤ 200 mV, thus demonstrating the increased CO tolerance of these three catalysts.

As an example, in ref. [86], the observed onset potential of CO oxidation below 0.4 V served as evidence of the improved CO tolerance of SnO_2_-supported Pt catalysts.

It is well accepted [10,87,88,89] that CO electrooxidation on Sn-containing catalysts is the result of a bifunctional mechanism in which OH_ads_ species are formed at Sn sites at much less positive potentials than on the active metal. Indeed, in an acidic medium OH_ads_ species are available on the surface of (i) Pt-based electrodes at E > 600 mV; (ii) Sn-containing electrodes at potentials E ≤ 100 mV [90,91].

CO_ads_ stripping voltammograms of the Pt/Ti_0.9_Sn_0.1_O_2_–C catalysts evidenced the presence of two overlapping electrooxidation peaks at 695 and 775–785 mV, as listed in Table 4. For the catalysts prepared using functionalized carbon (Pt/50FC and Pt/75FC), the second peak was observed at less positive potential (at 775 mV) compared to the position of this CO oxidation peak observed for the Pt/50C catalyst (at 785 mV).

Thus, the functionalization of the carbonaceous part of composite support materials results in only a small shift (ca. 10 mV) of the position of the second CO stripping peak in comparison to that obtained on the sample synthesized using unmodified carbon (Pt/50C). According to the literature, this kind of shift can be an indication of (i) the increase in the CO tolerance of the catalysts [67], as well as (ii) some extent of the agglomeration of Pt nanoparticles [92]. However, the TEM results shown in Figure 3 demonstrate the presence of well-dispersed, uniformly distributed Pt on the surface of all three catalysts. Thus, the shift of this peak on the FC-containing catalysts by ~10 mV toward less positive potential values may indicate a somewhat higher extent of CO tolerance due to better mixed oxide coating over carbon. It should be noted that on all three catalysts, the position of the first CO oxidation peak appeared at around 695 mV (see Figure 10 and Table 4).

According to our previous studies, the main CO_ads_ stripping peak on the reference Pt/C catalyst is located at ca. 795 mV [69], while on Sn–Pt/C alloy-type catalysts, the electrooxidation of CO occurs at about 700 mV [84].

In ref. [89], for the Pt/SnO*_x_*/C catalytic system, broadened and split into two components, CO oxidation peaks were demonstrated: the peak at higher potential was ascribed to CO oxidation on Pt, while the shoulder at lower potential (≤700 mV) was attributed to CO oxidation at the Pt–SnO*_x_* interface.

It should be noted that Li et al. [93] obtained much more positive values for both the E_CO,onset_ (707 mV) and the position of the maximum of the CO oxidation peak (839 mV) on the Pt/Ti_0.9_Sn_0.1_O_2_–C electrocatalyst.

As shown in Figure 10, the main difference in the shape of the CO_ads_ stripping voltammograms was observed in the so-called “pre-peak” region: the most pronounced “pre-peak” area was measured on the Pt/75FC catalyst with 75 wt.% of FC in the composite. It should be emphasized that among the studied Sn-containing samples, this catalyst has the most homogeneous oxide distribution on the carbon backbone, especially in terms of the general presence of tin species over the carbon. According to literature data [94,95] and our previous studies of Pt catalysts supported on Mo-containing composite [96], the oxidation of weakly adsorbed CO at certain sites of Pt in atomic proximity to the oxophilic dopant atoms was responsible for the appearance of the so-called “pre-peak”. Thus, CO_ads_ stripping voltammetry results, consistently with TEM and XPS observations, indicate that an increase in the content of functionalized carbon in the composite material leads to an increase in the amount of this type of catalytically active centers (in this case, Pt and Sn ensemble sites), responsible for increasing the activity of CO electrooxidation.

As can be seen from Figure 10, after 500-cycle stability testing, a slight shift (ca. 10–20 mV) of the second peak of CO electrooxidation towards less positive potential values was observed compared to the values obtained on fresh samples. However, it should be noted that the position of the first peak at 695 mV and the “pre-peak” region remain unchanged, demonstrating good stability of the sites responsible for the low-potential CO electrooxidation.

Thus, it can be concluded that the electrochemical behavior of the three studied electrocatalysts reflects the general characteristics of Pt/SnO*_x_*-containing systems, which are determined by the direct Sn–Pt interactions.

The influence of the use of FC, along with its amount in the Ti_0.9_Sn_0.1_O_2_–C composites on the performance of the Pt catalysts supported on them in the HOR and the ORR was depicted in Figure S6 in the Supplementary Materials. For comparison, the result obtained under similar conditions on a commercial reference catalyst 20 wt.% Pt/C was also included.

Figure S6a presents HOR voltammograms (positive-going scans) recorded by the RDE technique in H_2_-saturated 0.5 M H_2_SO_4_ at a rotation speed of 900 rpm. The results show that the diffusion-limiting current of the composite-supported catalysts is similar to that of the reference 20 wt.% Pt/C, reaching a limiting plateau at approximately 0.05 V (see Figure S6a). It is well known in the literature [97] that HOR on Pt-containing electrodes in an acid solution might be too fast to be measured by the RDE technique; consequently, distinguishing the catalytic activities of various Pt-based materials is difficult. Nonetheless, these findings indicate that all the tested catalysts exhibited good activity in the HOR.

Catalytic activity in the ORR of Pt electrocatalysts was investigated by RDE measurements in an O_2_-saturated 0.5 M H_2_SO_4_ solution (see Figure S6b). As mentioned in the Supplementary Materials, potential dynamic polarization curves were recorded at six different rotation speeds. Increasing current densities with increasing rotation speed demonstrated the expected effect of faster oxygen diffusion to the catalyst surface. To compare the activity of the investigated catalysts in the ORR, Figure S6b shows potential sweeps in the range of 0.3 and 1.0 V measured at 900 rpm. As can be seen from Figure S6b in the Supplementary Materials, very similar values of the E_ORR,onset_ for the ORR (E_ORR,onset_ ~ 965 ± 10 mV) and diffusion-limited current densities, where the reaction rate is limited by the O_2_ availability at the surface of the electrode, were achieved on the reference Pt/C and all studied composite supported catalysts, indicating high activity in this reaction.

It should be noted that, based on our experience with oxide-containing catalysts, as a consequence of the difference in saturation currents reflecting the difference in the mass transfer properties of the catalyst layers a slightly lower limiting current can be expected for the case of catalysts with high oxide content (75 wt.%) in composite supports [77]. However, as follows from Figure S6b, almost no difference was observed between the diffusion-limited current densities of composite-supported catalysts and the reference Pt/C. This can be explained both by the reduced oxide content in these composites (25 and 50 wt.%) and by the homogeneity of the mixed oxide coating over carbon.

In our recent study, the positive effect of the reductive pre-treatment on the stability and activity of Mo- and Sn-containing composite-supported Pt catalysts was already demonstrated [68,98].

Sn–Pt alloy formation during reduction treatment was observed for the Pt/Ti_0.8_Sn_0.2_O_2_–C catalyst, which was attributed to the atomic vicinity of Pt and Sn surface species and the thermodynamics of the Sn–Pt couple [68]. In addition, we demonstrated earlier excellent electrocatalytic properties of the alloy-type Pt_3_Sn/C electrocatalysts prepared using Controlled Surface Reactions in the ethanol [99], methanol [100], and CO electrooxidation along with improved activity in the ORR [84]. In this regard, it can be expected that the reductive pre-treatment of the catalyst will have a positive effect on its electrocatalytic behavior.

In order to evaluate the influence of the reductive treatment on the behavior of the catalysts synthesized using functionalized carbon materials, as part of the XPS measurements, the sample Pt/50FC was *in situ* treated in 100 mbar hydrogen at 300 °C for 1 h in the preparation chamber of the electron spectrometer, then its electrochemical properties were investigated.

**Figure 11:**
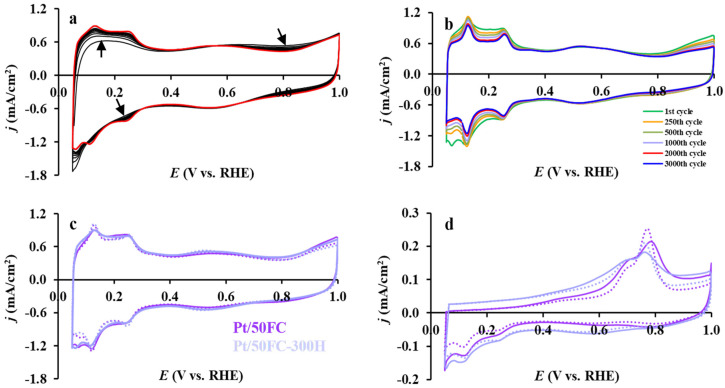
Cyclic voltammograms of the *as-prepared* Pt/50FC catalyst reduced at 300 °C (abbreviation in Table 4: Pt/50FC-300H). (**a**) CVs recorded during cleaning procedure between 2nd and 9th cycles in Ar-saturated solution (black curves) and 10th cycle (red curve). Arrows indicate the direction of changes; (**b**) CVs obtained on the Pt/50FC-300H sample during 3000-cycle stability test; (**c**) CVs and (**d**) CO_ads_ stripping voltammograms of the *as-prepared* (Pt/50FC (■)) and the reduced (Pt/50FC-300H (■)) catalysts. Recorded before (solid curves) and after 500 cycles (dotted curves) of the stability test with 100 mV s^−1^ (**a**–**c**) and 10 mV s^−1^ (**d**) sweep rate.

Our preliminary electrochemical experiments on the Pt/50FC-300H catalyst showed that reductive treatment has a positive effect on the stability and CO electrooxidation activity (see Table 4 and Figure 11).

The voltammograms shown in Figure 11a highlight the surface changes of the air-exposed Pt/50FC-300H catalyst observed during the first ten cycles of cyclic polarization between 50 and 1000 mV potential limits. In the first polarization cycles, the characteristic features of hydrogen adsorption/desorption on Pt become weakly expressed, probably due to the blocking of Pt sites by a thin surface SnO_x_ layer formed during the storage of the alloyed catalyst in air. This kind of dealloying into a Pt-rich core covered by a segregated SnO_x_ overlayer is a characteristic feature of Sn–Pt alloys exposed to an oxidizing ambient [84]. This overlayer is partially dissolved during cyclic polarization, which results in a gradual increase in the accessible Pt surface area, as shown in Figure 11a.

The change of the cyclic voltammograms obtained during the 3000-cycle stability test for the reduced Pt/50FC-300H catalyst is shown in Figure 11b. The ECSA_1_ and ΔECSA_N_ (N: 500 and 2500) values, calculated for this catalyst, are presented in Table 4. As shown in Table 4, the initial ECSA value of the *as-prepared* Pt/50FC and reduced Pt/50FC-300H catalysts was quite close (lying within the range of the experimental errors). In accordance with this, as can be seen from Figure 11c, the shape of the CVs obtained on the *as-prepared* and reduced catalysts was very similar (voltammograms overlapped each other). Thus, high-temperature reductive treatment, due to the good stabilizing effect of the oxide component of the composite, does not lead to the sintering of Pt nanoparticles. Moreover, the ECSA loss observed during the 500 and 2500-cycle stability tests was lower for the reduced Pt/50FC-300H catalyst compared to the *as-prepared* Pt/50FC counterpart, thus demonstrating its improved stability (see Table 4).

The CO_ads_ stripping voltammograms for the *as-prepared* Pt/50FC and reduced Pt/50FC-300H catalysts recorded before and after 500 cycles of the stability test are compared in Figure 11d. CO_ads_ stripping voltammograms mainly differed in the intensity of the “pre-peak” region, which was more pronounced on the Pt/50FC-300H catalyst, and in the shift of both CO electrooxidation peaks by approximately 10–20 mV toward less positive potential values on the Pt/50FC-300H catalyst, which were observed at 685 and 765 mV on this sample. It should be noted that the presence of a significant current in the “pre-peak” region preceding the main peak/peaks of CO oxidation is one of the most important indicators of increased low-potential CO electrooxidation activity.

It should also be emphasized that after a 500-cycle stability test, the position of the main CO electrooxidation peaks of the reduced catalyst remains unchanged. This fact serves as further evidence of the enhanced stability of the reduced catalytic systems.

Thus, based on these preliminary results, it can be concluded that pre-treatment of these catalytic systems in hydrogen can serve as an additional tool for improving the catalytic properties of tin-containing composite-supported Pt systems. However, it must be further emphasized that these are only preliminary results and systematic studies in this direction are required to determine the optimal temperature and duration for reductive pre-treatment.

## 4. Conclusions

The replacement of the carbon support with mixed oxide–carbon composite materials is a viable approach for enhancing the longevity and activity of Pt-based electrocatalysts designed for PEM fuel cells. This work explored the effect of the functionalization of the carbonaceous component on the structure of the mixed oxide and the influence of the structural properties of the composites on the electrochemical performance of the Pt catalysts deposited onto them. As model support systems, fine-grained Ti_0.9_Sn_0.1_O_2_ composited with unmodified and HNO_3_-glucose functionalized Black Pearls 2000 carbon were used. The effect of the amount of the mixed oxide was assessed by comparing the properties of catalysts prepared with composites with 50 and 75 wt.% functionalized carbon. Structural investigations with TEM/STEM/EDS elemental mapping revealed that the 10–20 nm mixed oxide particles of the composites with 50 wt.% carbon tended to form loose aggregates or chains around the perimeter of the unmodified carbon elements. On the contrary, much more homogeneously distributed mixed oxide particles appeared on the surface of the functionalized carbon, which can be attributed to the anchoring effect of the oxygen-containing functional groups. Decreasing the oxide content of the composite resulted in smaller, even more homogeneously distributed oxide particles. XPS studies confirmed the close coupling between the mixed oxide component of the composite and the Pt, which was dominated by the Sn–Pt interactions. Electrochemical studies confirmed the excellent CO electrooxidation properties of the catalysts, which is explained by the interplay of the Pt active metal particles and surface Sn species in their atomic vicinity. Similar reasons explained the good performance of the catalysts in the oxygen reduction reaction. The composite support with unmodified carbon significantly improved the long-term stability of the related electrocatalyst, but even better long-term behavior was experienced for catalysts on supports with functionalized carbon. The positive correlation between the uniformity of the oxide distribution over the carbon component and the stability of the catalysts demonstrates the importance of the oxide–Pt–carbon triple junctions in the electrocatalytic performance of the composite-supported catalysts.

## Figures and Tables

**Table 1:** Nominal composition and amount of precursor materials for preparation of the Ti_0.9_Sn_0.1_O_2_–C composites with the different oxide/C ratios using unmodified (C) and functionalized carbon (FC) materials.

Sample	Samples Nominal Composition ^a^	Sn Prec.^b^ (g)	TiO_2_ sol			Suspension of Carbon		
			H_2_O (mL)	HNO_3_ (mL)	Ti Prec.^c^ (mL)	C (g)	H_2_O (mL)	HNO_3_ (mL)
50C/50FC	50Ti_0.9_Sn_0.1_O_2_–50C	0.2016	15.65	1.75	1.53	0.50	12.5	0.876
75FC	25Ti_0.9_Sn_0.1_O_2_–75C	0.1008	7.82	0.88	0.77	0.75	24.1	1.752

^a^ Expected composition of composites with different Ti_0.9_Sn_0.1_O_2_/C mass ratio; ^b^ Sn precursor compound: tin (IV) chloride pentahydrate (SnCl_4_ × 5H_2_O, 98%); ^c^ Ti precursor compound: titanium-isopropoxide (Ti(O-*i-Pr*)_4_, 97%).

**Table 2:** Structural and surface composition characteristics of the Pt/50C, Pt/50FC and Pt/75FC electrocatalysts.

Sample	S_BET_, m^2^/g	Pt, nm TEM	Pt, wt.% XPS	Oxide/C, wt. Ratio, XPS	Ti/Sn, at. Ratio, XPS	Ti/Sn, at. Ratio, EDS
Pt/50C	n.m. ^a^	2.4±0.3	27.6	46/54	2.3:1	9.7:1
Pt/50FC	327	2.5±0.4	28.0	57/43	2.1:1	10.0:1
Pt/75FC	598	2.4±0.3	47.8	34/66	3.6:1	11.0:1

^a^ n.m.: not measured.

**Table 3:** Pt 4f_7/2_, Sn 3d_5/2_ and Ti 2p_3/2_ binding energies of the 20 wt.% Pt/Ti_0.9_Sn_0.1_O_2_–C catalysts synthesized using unmodified and functionalized carbon materials in the as-*prepared* (air exposed) state and after hydrogen exposure. The energy scale was referenced to the strongest peak of the graphitic carbon envelope at 284.4 eV [74].

Core Level	Pt/50C		Pt/50FC		Pt/75FC		Assignment
	Initial State	H_2_ Exp. ^a^	Initial State	H_2_ Exp. ^a^	Initial State	H_2_ Exp. ^a^	
Pt 4f_7/2_	71.3 (91%) 72.6 (6%) 75.1 (3%)	71.1 (100%)	71.3 (91%) 72.6 (7%) 75.0 (2%)	71.1 (100%)	71.3 (93%) 72.6 (5%) 75.1 (2%)	71.1 (100%)	metallic Pt Pt^2+^: PtO, Pt(OH)_2_ Pt^4+^: PtO_2_
Sn 3d_5/2_	484.9 (2%) 487.1 (98%)	484.8 (7%) 487.0 (93%)	484.6 (<1%) 487.1 (99%)	484.8 (5%) 487.0 (95%)	484.9 (<1%) 487.0 (99%)	484.9 (12%) 487.0 (88%)	metallic Sn Sn^4+^: SnO_2_
Ti 2p_3/2_	459.0	459.2	459.0	459.1	458.9	459.1	Ti^4+^: TiO_2_
C 1s ^b^	284.4	284.4	284.4	284.4	284.4	284.4	graphitic C

^a^ Hydrogen exposure was performed in 100 mbar H_2_ at 100 °C for 1 h in the high-pressure chamber of the electron spectrometer; ^b^ Strongest component of the graphitic carbon envelope.

**Table 4:** Electrochemical performance of the 20 wt.% Pt/Ti_0.9_Sn_0.1_O_2_–C catalysts synthesized using unmodified and functionalized carbon materials.

Sample ID	E_CO,max_, ^a^ mV	ECSA_1_, m^2^/g_Pt_^b^	ΔECSA_500_, [A] % ^c^	ΔECSA_2500_, [B] % ^c^	ΔECSA_10,000_, [C] % ^c^	[C−B] % ^d^
Pt/25C ^e^	785 (sh: 685)	58.5 ± 1.4	7.3	24.2	42.7	18.5
Pt/50C	785 (sh: 695)	56.4 ± 2.4	10.2	24.1	39.6	15.5
Pt/50FC	775 (sh: 695)	50.2 ± 1.7	6.1	28.0	34.4	6.4
Pt/50FC-300H ^f^	765 (sh: 685)	50.7 ± 2.1	5.7	21.0	n.m.	-
Pt/75FC	775 (sh: 695)	55.5 ± 1.6	8.3	29.9	37.3	7.4
Pt/C ^e^	795	87.2 ± 2.3	12.7	25.3	47.8	22.5

^a^ Potential of the main CO stripping peak of the fresh catalysts; ^b^ The average ECSA_1_ value obtained on fresh catalysts; ^c^ ΔECSA_N_ (N: 500, 2500 and 10,000): ECSA decrease after N cycles of the long-term stability test calculated according to the Equation (1); ^d^ [C−B] = [ΔECSA_10,000_ − ΔECSA_2500_]: changes in ΔECSA observed between 2500 and 10,000 cycles; ^e^ From ref. [66]; ^f^ Reductive pre-treatment at 300 °C; n.m.: not measured; sh = shoulder.

## Data Availability

The data presented in this study are available on request from the corresponding authors.

## Supplementary Materials

The following supporting information can be downloaded at: https://www.mdpi.com/article/10.3390/nano15050342/s1, Figure S1: Flow chart for the carbon functionalization performed using a two-step treatment with HNO_3_ and glucose; Figure S2: DTG curve of the carbon material functionalized with HNO_3_ and glucose; Figure S3: Ti–Sn, Sn–Pt elemental distributions and overlaps of the HAADF images with the Sn distribution for: (a) Pt/50C, (b) Pt/50FC and (c) Pt/75FC; Figure S4: TEM and high-resolution TEM images of the Pt/75FC electrocatalyst; Table S1: Results of nitrogen adsorption measurements of the composite materials and related Pt electrocatalysts; Figure S5: Nitrogen physisorption isotherms of 50C and 50FC composite materials along with Pt/50FC catalyst; Figure S6: Electrochemical characterization of the Sn-containing Pt electrocatalysts by RDE measurements at 900 rpm: Pt/50C, Pt/50FC and Pt/75FC. Results obtained on the reference Pt/C were included for comparison.

## Abbreviations

The following abbreviations are used in this manuscript:

MDPI	Multidisciplinary Digital Publishing Institute
DOAJ	Directory of open access journals
XRD	X-ray diffraction
TEM	Transmission electron microscopy
STEM	Scanning transmission electron microscopy
EDS	Energy-dispersive X-ray spectroscopy
HAADF	High angle annular dark field (Scanning transmission electron microscopy)
HRTEM	High-resolution transmission electron microscopy
XPS	X-ray photoelectron spectroscopy
PEM	Polymer electrolyte membrane
OFG	Oxygen-containing functional group
ORR	Oxygen reduction reaction
HOR	Hydrogen oxidation reaction
TG	Thermogravimetric analysis
BET	Brunauer–Emmett–Teller
CV	Cyclic voltammetry
GC	Glassy carbon
RHE	Reversible hydrogen electrode
ECSA	Electrochemically active surface area
RDE	Rotating disc electrode
JCPDS	Joint Committee on Powder Diffraction Standards
