# Supplementary material for: Advanced Pt/Ti(1−x)SnxO2–C Composite Supported Electrocatalyst with Functionalized Carbon for Sustainable Energy Conversion Technologies
Source: Nanomaterials (Basel). 2025 Feb 22;15(5):342. doi: 10.3390/nano15050342 (PMC11902096; doi:10.3390/nano15050342)
Supplement: Supplementary file 1 [file nanomaterials-15-00342-s001.zip › nanomaterials-3470310-supplementary.pdf]

SUPPLEMENTARY MATERIALS

Advanced Pt/$Ti_{(1-x)}Sn_xO_2$–C Composite Supported Electrocatalyst with Functionalized Carbon for Sustainable Energy Conversion Technologies

Cristina Silva [1,2], Zoltán Pászti [1,*], Khirdakhanim Salmanzade [1], Dániel Olasz [3], Erzsébet Dodony [3], György Sáfrán [3], Ágnes Szegedi [1], Zoltán Sebestyén [1], András Tompos [1,*] and Irina Borbáth [1]

[1] Institute of Materials and Environmental Chemistry, HUN-REN Research Centre for Natural Sciences,
Magyar Tudósok Körútja 2, H-1117 Budapest, Hungary; silva.cristina@ttk.hu (C.S.); ksalmanzade5@gmail.com (K.S.); szegedi.agnes@ttk.hu (Á.S.); sebestyen.zoltan@ttk.hu (Z.S.);
borbath.irina@ttk.hu (I.B.)
[2] Department of Physical Chemistry and Materials Science, Faculty of Chemical Technology and
Biotechnology, Budapest University of Technology and Economics, Műegyetem rkp. 3, H-1111 Budapest, Hungary
[3] Institute for Technical Physics and Materials Science, Centre for Energy Research, Konkoly-Thege Miklós út 29-33, H-1121 Budapest, Hungary; olasz.daniel@ek.hun-ren.hu (D.O.);
dodony.erzsebet@ek.hun-ren.hu (E.D.); safran.gyorgy@ekhun-ren.hu (G.S.)
* Correspondence: paszti.zoltan@ttk.hu (Z.P.); tompos.andras@ttk.hu (A.T.); Tel.: +36-1-3826-412 (Z.P.);
+36-1-3826-501 (A.T.)

1. Materials and Methods

1.1. Details of the functionalization of commercial carbon

The functionalization of commercial Black Pearls 2000 carbon was performed according to the procedure described in our recent study [1]. In this work, commercial carbon pre-treated in nitrogen at 1000 °C was functionalized by a two-step treatment with HNO_3 and glucose. The flow chart of the carbon functionalization procedure involving a two-step treatment with HNO_3 and glucose is shown in Figure S1.

<u>Two-step treatment with HNO_3 and glucose (HNO_3 + glucose):</u>

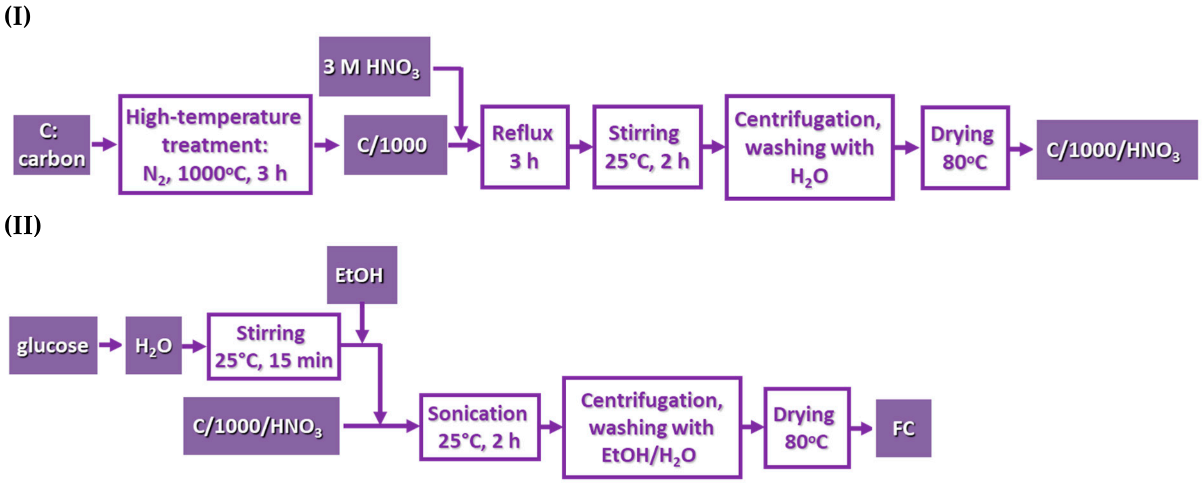

Figure S1. Flow chart for the carbon functionalization performed using a two-step treatment with HNO_3 and glucose.

Preparation

1) ***Pre-treatment in nitrogen at 1000 °C (C1000)***: A quartz reactor with carbon powder was purged with N_2 (flow rate= 8.5 ml/min) at room temperature (RT) for 2 hours to eliminate oxygen. After that high-temperature treatment (HTT) in N_2 flow was done using the following experimental parameters: heating rate= 10 °C/min, N_2 flow rate= 8.5 ml/min, final temperature= 1000 °C. Carbon was kept at 1000 °C for 3 h then the reactor was cooled down in flowing N_2 to RT.
2) ***Surface modification with HNO_3:*** 500 mg of the C1000 carbon was refluxed in 100 ml of a 3 M HNO_3 solution for 3 h. The mixture was then cooled to room temperature and stirred for another 2 h. After this, the resulting suspension was centrifuged and washed three times with distilled water. Finally, the samples were dried in an oven at 80-90 °C for 24 hours.
3) ***Glucose doped carbon:*** Glucose (1 g) was dissolved in 20 ml of water; after complete dissolution, 80 ml of ethanol was added to the mixture and the system was stirred for 5 minutes to ensure complete homogeneity. 500 mg of the C1000/HNO_3 carbon was placed into the 250 ml beaker. Glucose in an ethanol/water solution was added to carbon and the suspension was sonicated at room temperature for 2 h.

   Washing: the centrifuge tube was filled with the carbon suspension and suspension was centrifuged for 10 minutes. The separated transparent liquid was removed and an alcohol solution was added. After that, a magnetic stirrer was installed and then the suspension was stirred for 5 minutes and centrifuged for 10 minutes (washing with alcohol solution was performed 3 times).

   Drying: the material was dried at 80-90 °C overnight.

1.2. Physicochemical characterization of the composite support materials and the electrocatalysts

Thermogravimetric (TG) measurements were done using a modified Perkin-Elmer (Waltham, MA, USA) TGS-2 thermobalance [1]. About 3 mg samples were measured in inert argon atmosphere at a flow rate of 140 ml min^{-1}. The samples were heated at a rate of 20 °C min^{-1} from room temperature to the final temperature of 900 °C in a platinum sample pan.

X-ray powder diffraction (XRD) patterns were obtained using a Philips (Eindhoven, the Netherlands) model X'PERT MPD and PW 3710 based PW 1050 Bragg–Brentano parafocusing goniometer with CuKα radiation, graphite monochromator and proportional counter.

Nitrogen physisorption measurements were carried out at temperature of liquid nitrogen using Thermo Scientific Surfer automatic volumetric adsorption analyzer (Thermo Fischer Scientific, Berlin, Germany). The specific surface area was calculated by the BET method in the range of relative pressures from 0.05 to 0.30.

Microstructure was investigated by Transmission Electron Microscopy (TEM) by means of a FEI (Hillsboro, OR, USA) Titan Themis 200 kV C_s—corrected microscope with 0.09 nm HRTEM resolution. The scanning transmission electron microscopy (STEM) capabilities of the instrument were utilized in recording of high resolution elemental maps by the Energy Dispersive Spectroscopy (EDS) technique. Pt particle distribution was determined by measuring the diameters of at least 800 randomly selected metal particles in five micrographs of each sample taken from non-aggregated areas. Evaluation and processing of the high resolution micrographs was performed using the ImageJ software [2].

X-ray photoelectron spectroscopy (XPS) measurements were performed using an electron spectrometer manufactured by OMICRON Nanotechnology GmbH (Taunusstein, Germany). MgKα (1253.6 eV) radiation was used as excitation source and data were acquired with 1 eV spectral resolution (30 eV pass energy). The powdered composite supports and catalysts were suspended in isopropanol and drops of this suspension were dried onto stainless steel sample plates. Spectra were processed with the CasaXPS package [3] as described in our previous work [4]. Shortly, the metallic Pt 4f contributions were modeled by a $4f_{7/2}$-$4f_{5/2}$ doublet with asymmetric line shape while ionic Pt signals were taken into account by weak doublets with symmetric peaks. C 1s spectra were fitted by a combination of an asymmetric graphitic line shape and additional symmetric peaks arising from heteroatom-bound carbon species. Tin 3d spectra were fitted by $3d_{5/2}$-$3d_{3/2}$ doublets after correction for satellite peaks

arising from the Sn $3d_{3/2}$ peak by $MgK\alpha_{3,4}$ excitation. Quantitative evaluation of the data was performed with the XPSMultiQuant package [5,6], during which a homogeneous depth distribution was assumed for all components as described in our previous studies. Chemical states were identified using the NIST database [7], the publication [8] or other literature as indicated. Binding energies were related to the lowest binding energy contribution of the C 1s envelope, which was assigned to graphite-like (sp^2-hybridized) carbon in the carbonaceous backbone (284.4 eV).

2.5. Electrochemical characterization

Electrocatalytic performance of the Pt electrocatalysts was studied by cyclic voltammetry (CV) and CO_{ads}-stripping voltammetry measurements combined with stability test involving 500 polarization cycles and the second CO_{ads}-stripping voltammetry measurement (for more details see refs. [9,10]). In the long-term stability test, the samples were submitted to cyclic polarization at a 100 mV s^{-1} scan rate for 10,000 cycles between 50 and 1000 mV potential limits.

The measurements were performed in a conventional three-electrode electrochemical glass cell using a Biologic (Seyssinet-Pariset, France) SP150 potentiostat and the EC-LAB software package. The working glassy carbon (GC) electrode (d= 0.3 cm, geometric surface area A= 0.0707 cm^2) was polished before each test to remove any traces of organic impurities. The catalyst ink was prepared using the standard method described in our previous work [10]; the Pt loading of the electrodes was 10 μg cm^{-2}. The reference electrode was reversible hydrogen electrode (RHE) and Pt was used as counter electrode. All potentials are given on RHE scale.

The electrochemically active surface area (ECSA) of the electrocatalysts was determined from the charge needed for oxidation of the under potentially deposited hydrogen [11], using conventional baseline correction as described in our previous study [12].

The change of the electrochemically active surface area of Pt during the stability test after N cycles of polarization is presented as $ECSA_N$ normalized to $ECSA_1$ measured in the 1st cycle on the same sample ($ECSA_N/ECSA_1$). Thus, the loss in ECSA during N-cycle stability test ($\Delta ECSA_N$) was calculated from the charges originated from the hydrogen desorption in the 1st and N^{th} cycles according to the Equation 1 [12]:

$$\Delta ECSA_N = \{1-(ECSA_N/ECSA_1)\}\times 100\% \qquad (1)$$

Electrochemical performance of Sn-containing Pt electrocatalysts was compared with commercial reference 20 wt.% Pt/C (Quintech).

Catalytic activity of the catalysts was also tested by rotating disc electrode (RDE) technique in the oxygen reduction reaction and in the hydrogen oxidation reaction as described in our previous study [13]. The measurements were carried out in O_2 or H_2 saturated 0.5 M H_2SO_4 electrolyte. The diameter of RDE electrode and Pt loading used in these experiments was the same as during CV measurements. In the ORR, polarization curves were recorded by cathodic scan rotating the electrode at 225, 400, 625, 900, 1225 and 1600 revolutions min^{-1} (rpm). Catalytic activity in the HOR was investigated at 400, 625, 900, 1225 and 1600 rpm.

2. Results and discussion

2.1. Characterization of the functionalized carbon materials by thermogravimetric (TG) measurements and XPS

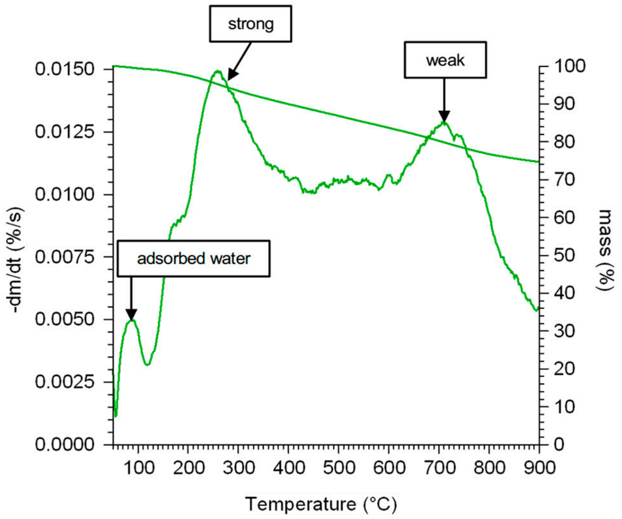

Figure S2. DTG curve of the carbon material functionalized with HNO_3 and glucose.

Thermal analysis is a straightforward method for assessing the nature of the introduced functional groups. According to the TG results after two-step treatment with HNO_3 and glucose, the content of oxygen-containing surface functional groups was 25.3 wt.%.

As shown in Figure S2, the thermal decomposition of functionalized carbon (FC) proceeds with elimination of the surface functional groups in two steps. According to the literature [14, 15] during TG measurements done on glucose-doped carbon materials the mass losses observed at temperature below 500 °C were assigned to the pyrolysis of glucose, which proceeds with elimination of water and volatile decomposition products.

Moreover, it has been reported in our previous study that stronger acid groups (e.g. carboxylic, anhydride groups) decompose between 200 and 500 °C with maximum at 273 °C, whereas the decomposition of the weaker acid sites (e.g. lactone, phenol, carbonyl groups, etc.) started at higher temperatures (407 °C).

The composition of the functionalized carbon materials was determined using XPS measurements (C= 86.6 and O= 13.4 atom%). Moreover, the high density of -OH groups was evidenced by XPS.

2.2. Characterization by TEM

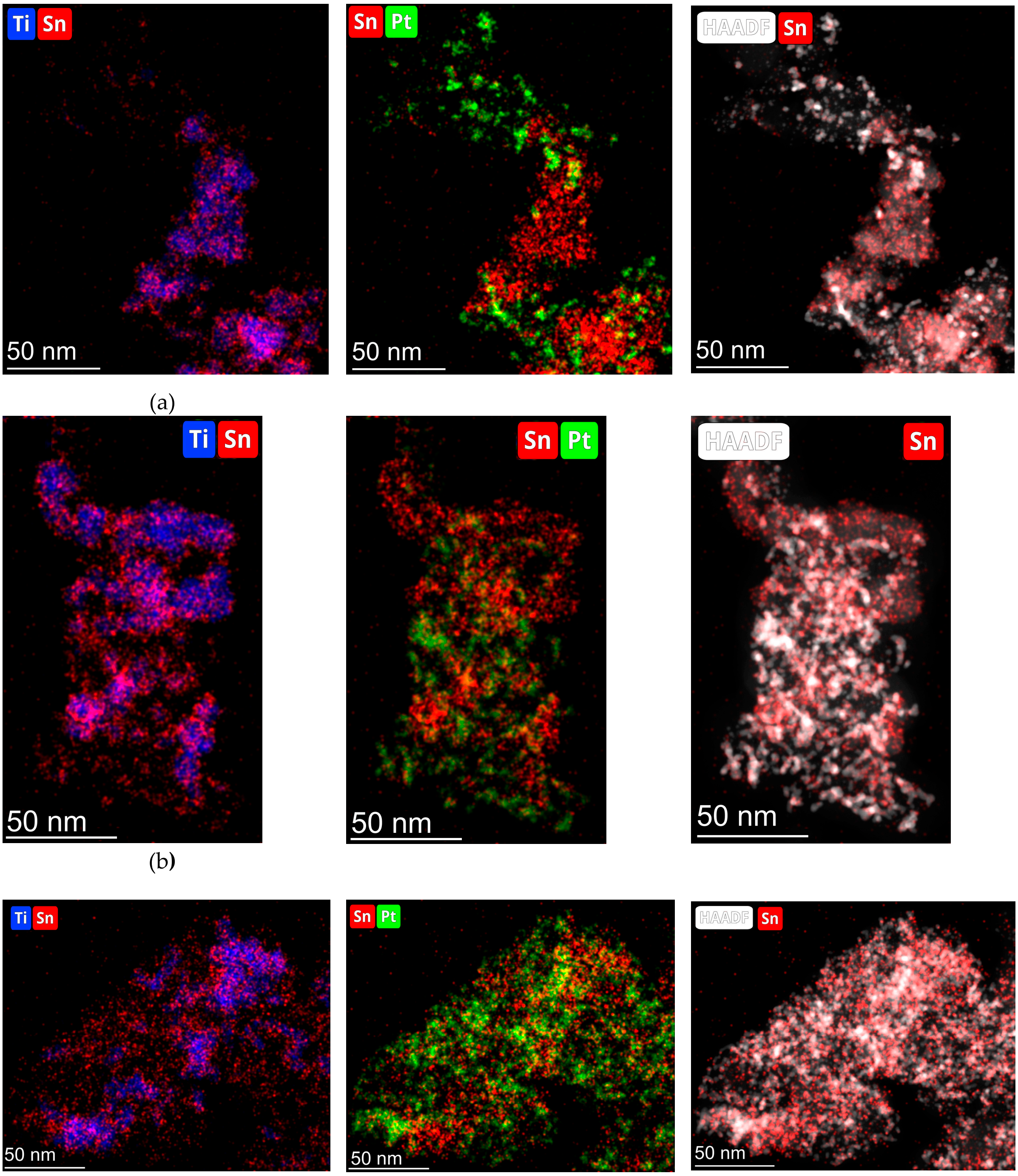

(c)

Figure S3. Ti-Sn, Sn-Pt elemental distributions and overlaps of the HAADF images with the Sn distribution for: (a) Pt/50C, (b) Pt/50FC and (c) Pt/75FC.

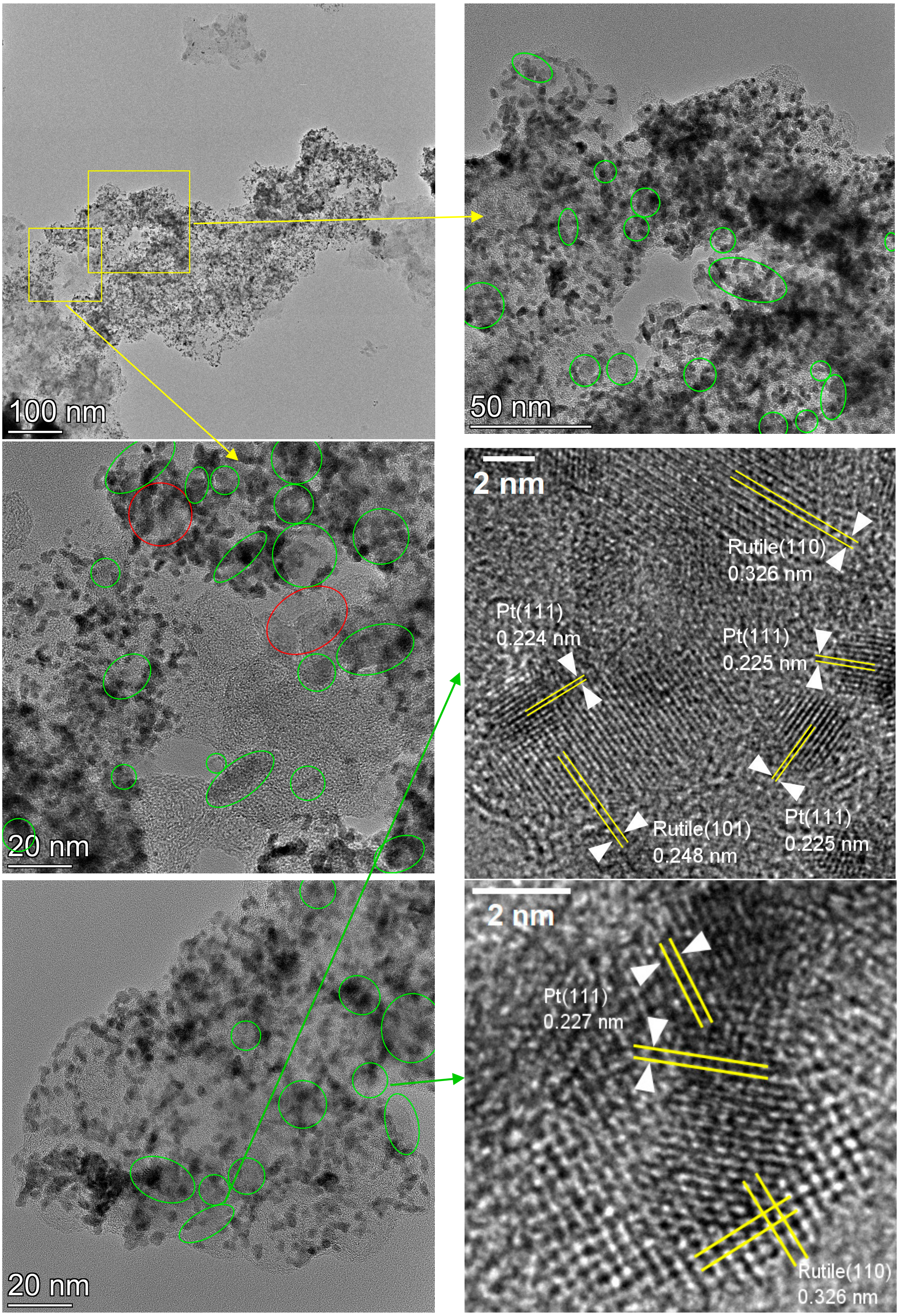

Figure S4. TEM and high resolution TEM images of the Pt/75FC electrocatalyst. Individual oxide crystals are encircled in green, small agglomerations of oxide crystals are indicated by red.

2.3. Characterization by nitrogen physisorption measurements

Table S1. Results of nitrogen physisorption measurements of composite materials and related Pt electrocatalysts.

Samples	S_{BET}, m²/g	Total pore volume, cm³/g	Pore diameter nm	Mesopore surface area, m²/g	Micropore volume cm³/g
50C	675	0.905	22.9	350	0.176
50FC	443	0.594	12.0	295	0.095
75FC	621	0.700	12.9	332	0.165
Pt/50FC	327	0.470	11.2	209	0.073
Pt/75FC	598	0.670	12.1	322	0.161

The N_2 adsorption isotherms are characteristic of composite materials and related Pt electrocatalysts composed of a micro/mesoporous carbon and a mesoporous metal oxide component (Figure S4). The isotherms basically are of mixture of type II and IV, exhibiting two distinct hysteresis loops. The steep rise at very low relative pressures is in connection with micropore filling with condensed liquid nitrogen. The narrow H3-type hysteresis loop, closing at a relative pressure of 0.42, is typical of activated carbon with slit-like pores, whereas the H2-type loop at higher relative pressures is indicative of mesoporous titania materials. The H3 hysteresis loops are very flat, showing the rather microporous character of the carbon support and demonstrate a wide pore size distribution in a non-uniform pore system. The metal oxide part with H2 type hysteresis loop however gives a 11-13 nm pore size maximum for all the functionalized carbon-containing materials (see Table S1).

The non-functionalized carbon-containing composite with 50 wt.% carbon content (50C) has bigger mesopores and higher micropore volume, therefore a somewhat bigger specific surface area and pore volume is calculated (see Table S1). However, comparing its hysteresis loop with the 50FC sample it shows a slightly narrower pore size distribution (Figure S5). It can be concluded that the functionalization of carbon results not only in the change of carbon pore structure, but the morphology of inorganic component is also changed probably due to the different interaction of the oxide with the functionalized, more hydrophilic surface of carbon.

Pt loading resulted in some decrease of surface area, pore volume and pore diameter due to pore blocking effect of platinum nanoparticles. As shown in Table S1, a more pronounced decrease of these parameters after Pt loading was observed on the Pt/50FC catalyst with a lower content of functionalized carbon. Electron microscopy studies have revealed that increasing the amount of FC component in the composite from 50 to 75 wt.% results in a significant decrease in the particle size of the mixed oxide and an even more uniform distribution of the oxide on the functionalized carbonaceous material.

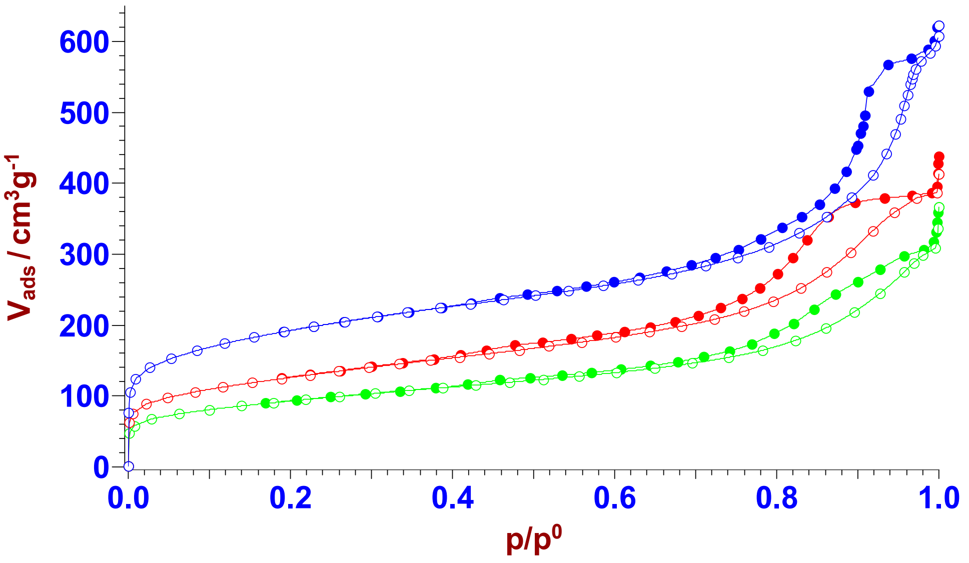

Figure S5. Nitrogen physisorption isotherms of 50C (blue line), 50FC catalysts (red line) and Pt/50FC catalyst (green line).

2.4. Electrochemical characteristics of the electrocatalysts

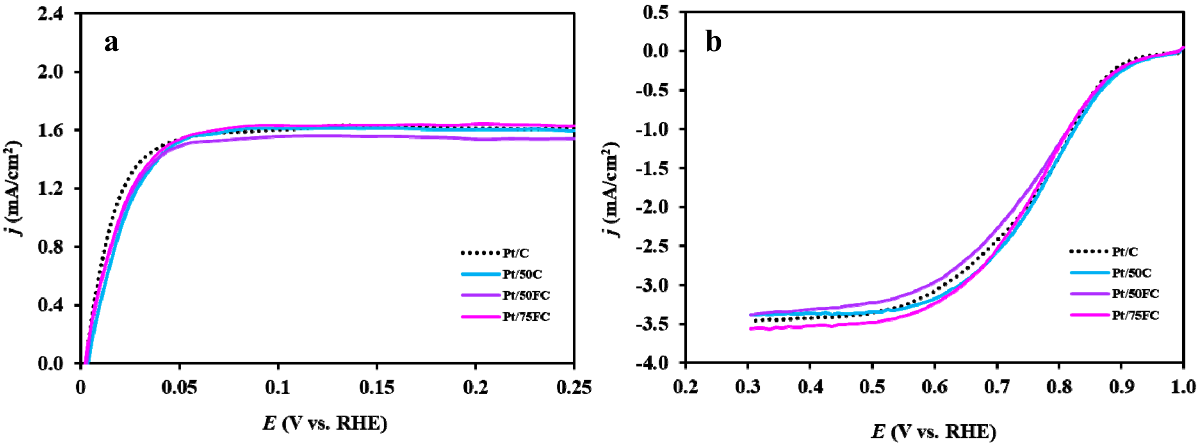

Figure S6. Electrochemical characterization of the Sn-containing Pt electrocatalysts by RDE measurements at 900 rpm: Pt/50C (■), Pt/50FC (■) and Pt/75FC (■). Results obtained on the reference Pt/C were included for comparison (■). (a) HOR curves obtained in a H_2-saturated 0.5 M H_2SO_4; (b) ORR curves obtained in O_2-saturated 0.5 M H_2SO_4. Recorded at 10 mV s^{-1}, T= 25 °C. The currents were normalized to the geometric area of the glassy carbon electrode.

References

[1] Borbáth, I.; Zelenka, K.; Vass, Á.; Pászti, Z.; Szijjártó, G.P.; Sebestyén, Z.; Sáfrán, G.; Tompos, A. CO Tolerant Pt Electrocatalysts for PEM Fuel Cells with Enhanced Stability against Electrocorrosion. *Int. J. Hydrogen Energy* **2021**, *46*, 13534–13547, doi:10.1016/j.ijhydene.2020.08.002, and references cited therein.

[2] Rasband, W.S. ImageJ (Version 1.54f) Available online: https://imagej.nih.gov/ij/. Accessed on 20 January 2025.

[3] N. Fairley CasaXPS: Spectrum Processing Software for XPS, AES and SIMS SIMS (Version 2.3.12) Available online: http://www.casaxps.com. Accessed on 20 January 2025.

[4] Silva, C.; Salmanzade, K.; Borbáth, I.; Dódony, E.; Olasz, D.; Sáfrán, G.; Kuncser, A.; Pászti-Gere, E.; Tompos, A.; Pászti, Z. Reductive Treatment of Pt Supported on $Ti_{0.8}Sn_{0.2}O_2$-C Composite: A Route for Modulating the Sn–Pt Interactions. *Nanomaterials* **2023**, *13*, doi:10.3390/nano13152245.

[5] Mohai, M. XPS MultiQuant: Multimodel XPS Quantification Software. Surf. Interface Anal. 2004, 36, 828–832, doi:10.1002/sia.1775.
[6] Mohai, M. XPS MultiQuant: Multi-Model X-Ray Photoelectron Spectroscopy Quantification Program, (Version 7.83) Available online: http://aki.ttk.mta.hu/XMQpages/XMQhome.php. Accessed on 20 January 2025.
[7] Naumkin, A.V.; Kraut-Vass, A.; Gaarenstroom, S.W.; Powell, C.J. NIST X-ray Photoelectron Spectroscopy Database, Version 5.0. Available online: http://srdata.nist.gov/xps/. Accessed on 21 February 2025.
[8] Moulder, J.F.; Stickle, W.F.; Sobol, P.E.; Bomben, K.D. Handbook of X-Ray Photoelectron Spectroscopy; Perkin-Elmer Corp. Eden Prairie, 1992; ISBN 0-9627026-2-5.
[9] Borbáth, I.; Salmanzade, K.; Pászti, Z.; Kuncser, A.; Radu, D.; Neaţu, Ş.; Tálas, E.; Sajó, I.E.; Olasz, D.; Sáfrán, G.; Szegedi, Á.; Florea, M.; Tompos, A. Strategies to Improve CO Tolerance and Corrosion Resistance of Pt Electrocatalysts for Polymer Electrolyte Membrane Fuel Cells: Sn-Doping of the Mixed Oxide–Carbon Composite Support. *Catal. Today* **2024**, *438*, 114788, doi:10.1016/j.cattod.2024.114788
[10] Borbáth, I.; Tálas, E.; Pászti, Z.; Zelenka, K.; Ayyubov, I.; Salmanzade, K.; Sajó, I.E.; Sáfrán, G.; Tompos, A. Investigation of Ti-Mo Mixed Oxide-Carbon Composite Supported Pt Electrocatalysts: Effect of the Type of Carbonaceous Materials. *Appl. Catal. A Gen*. **2021**, *620*, doi:10.1016/j.apcata.2021.118155.
[11] Woods, R. Electroanalytical Chemistry: A Series of Advances: Volume 9. In; Bard, A.J., Ed.; Dekker Marcel Inc: New York, 1976; pp. 1–162 ISBN 9780824764289.
[12] Gubán, D.; Borbáth, I.; Pászti, Z.; Sajó, I.; Drotár, E.; Hegedus, M.; Tompos, A. Preparation and Characterization of Novel $Ti_{0.7}W_{0.3}O_2$-C Composite Materials for Pt-Based Anode Electrocatalysts with Enhanced CO Tolerance. *Appl. Catal. B Environ*. **2015,** *174–175*, 455–470, doi:10.1016/j.apcatb.2015.03.031.
[13] Ayyubov, I.; Tálas, E.; Salmanzade, K.; Kuncser, A.; Pászti, Z.; Neaţu, Ş.; Mirea, A.G.; Florea, M.; Tompos, A.; Borbáth, I. Electrocatalytic Properties of Mixed-Oxide-Containing Composite-Supported Platinum for Polymer Electrolyte Membrane (PEM) Fuel Cells. *Materials* (Basel). **2022**, *15*, 3671, doi:10.3390/ma15103671
[14] Odetola, C.; Trevani, L.; Easton, E.B. Enhanced activity and stability of Pt/TiO_2/carbon fuel cell electrocatalyst prepared using a glucose modifier. *J. Power Sources* **2015**, *294*, 254-263. http://doi.org/10.1016/j.jpowsour.2015.06.066
[15] Odetola, C.; Easton, E.B.; Trevani, L. Investigation of TiO_2/carbon electrocatalyst supports prepared using glucose as a modifier. *Int. J. Hydrogen Energy* **2016**, *41*, 8199-8208. http://doi.org/10.1016/j.ijhydene.2015.10.035
